# Supplementary material for: Design, Synthesis and Biological Evaluation of Novel Phenyl-Substituted Naphthoic Acid Ethyl Ester Derivatives as Strigolactone Receptor Inhibitor
Source: Int J Mol Sci. 2024 Mar 31;25(7):3902. doi: 10.3390/ijms25073902 (PMC11012203; doi:10.3390/ijms25073902)
Supplement: Supplementary file 1 [file ijms-25-03902-s001.zip › ijms-2915918-supplementary.pdf]

Supporting Information

Design, Synthesis and Biological Evaluation of Novel Phenyl-Substituted Naphthoic Acid Ethyl Ester Derivatives as Strigolactone Receptor Inhibitor

Lin Du [1], Xingjia Li [2], Yimin Ding [1], Dengke Ma [1], Chunxin Yu [2], Hanqing Zhao [2], Ye Wang [2], Ziyan Liu [2] and Liusheng Duan [1,2,*]

[1] State Key Laboratory of Plant Physiology and Biochemistry, Engineering Research Center of Plant Growth Regulator, Ministry of Education & College of Agronomy and Biotechnology, China Agricultural University, Beijing 100193, China; s20183101661@cau.edu.cn (L.D.); ymyf12345678@163.com (Y.D.); mdk@cau.edu.cn (D.M.)

[2] College of Plant Science and Technology, Beijing University of Agriculture, Beijing 102206, China; li1257391684@163.com (X.L.); cxyu0303@foxmail.com (C.Y.); zhaohanqingbua@163.com (H.Z.); wangyebua@126.com (Y.W.); liuziyan8028@163.com (Z.L.)

* Correspondence: duanlsh@cau.edu.cn; Tel.: +86-10-62731301

Contents

The Spectra of ^{1}H, ^{13}C and HRMS...3-29

Molecular docking results of C6 and DL1b with the SL receptor protein ShHTL7 (PDB code: 7SNU) 27

Supplementary methods..31-32

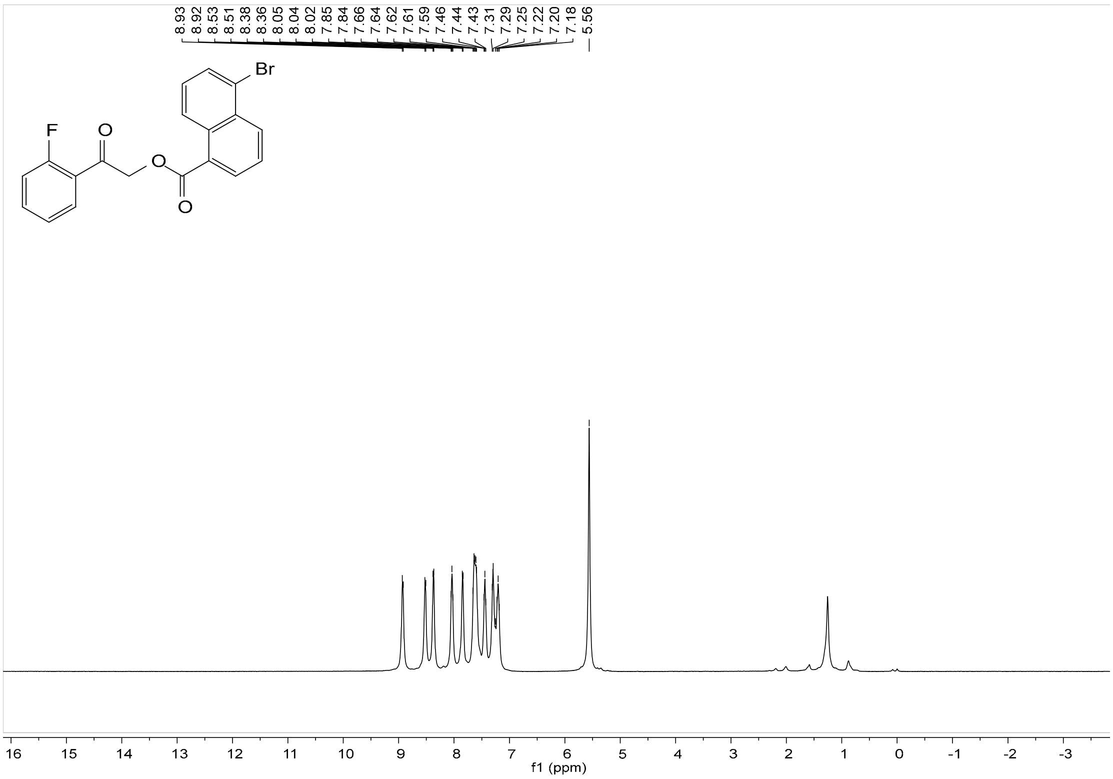
8.93
8.92
8.53
8.51
8.38
8.36
8.05
8.04
8.02
7.85
7.84
7.66
7.64
7.62
7.61
7.59
7.46
7.44
7.43
7.31
7.29
7.25
7.22
7.20
7.18
5.56
Br
F
O
O
O
16 15 14 13 12 11 10 9 8 7 6 5 4 3 2 1 0 -1 -2 -3
f1 (ppm)

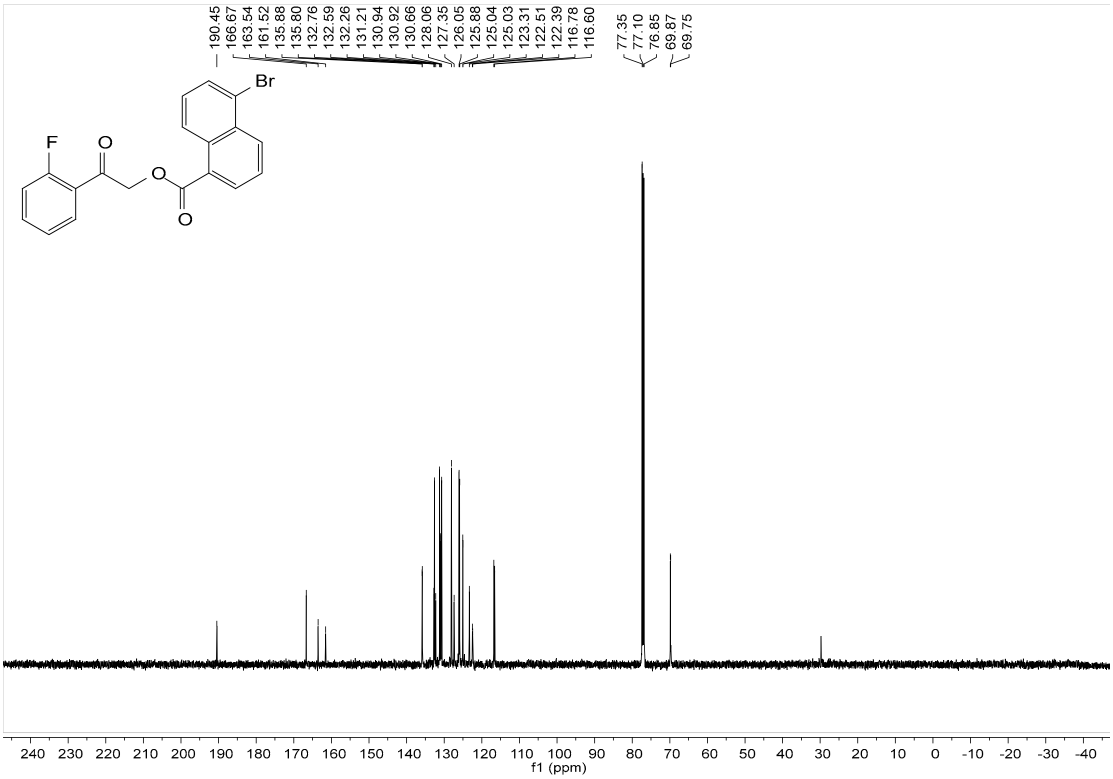
190.45
166.67
163.54
161.52
135.88
135.80
132.76
132.59
132.26
131.21
130.94
130.92
130.66
128.06
127.35
126.05
125.88
125.04
125.03
123.31
122.51
122.39
116.78
116.60
77.35
77.10
76.85
69.87
69.75
Br
F
O
O
O
240 230 220 210 200 190 180 170 160 150 140 130 120 110 100 90 80 70 60 50 40 30 20 10 0 -10 -20 -30 -40
f1 (ppm)

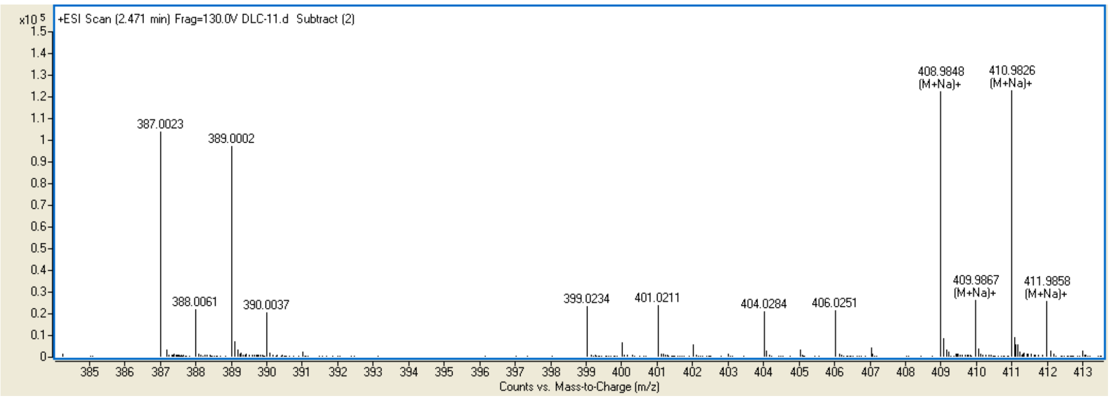

Figure S1. ^{1}H, ^{13}C and HRMS spectra of **C1**

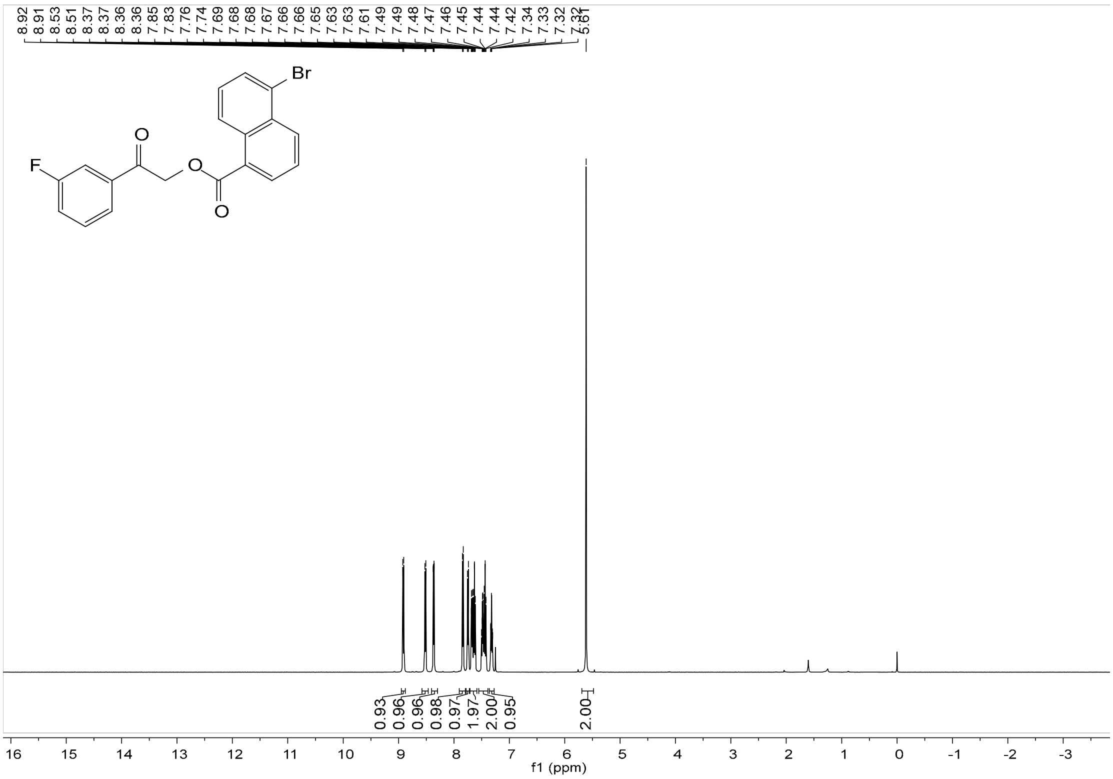

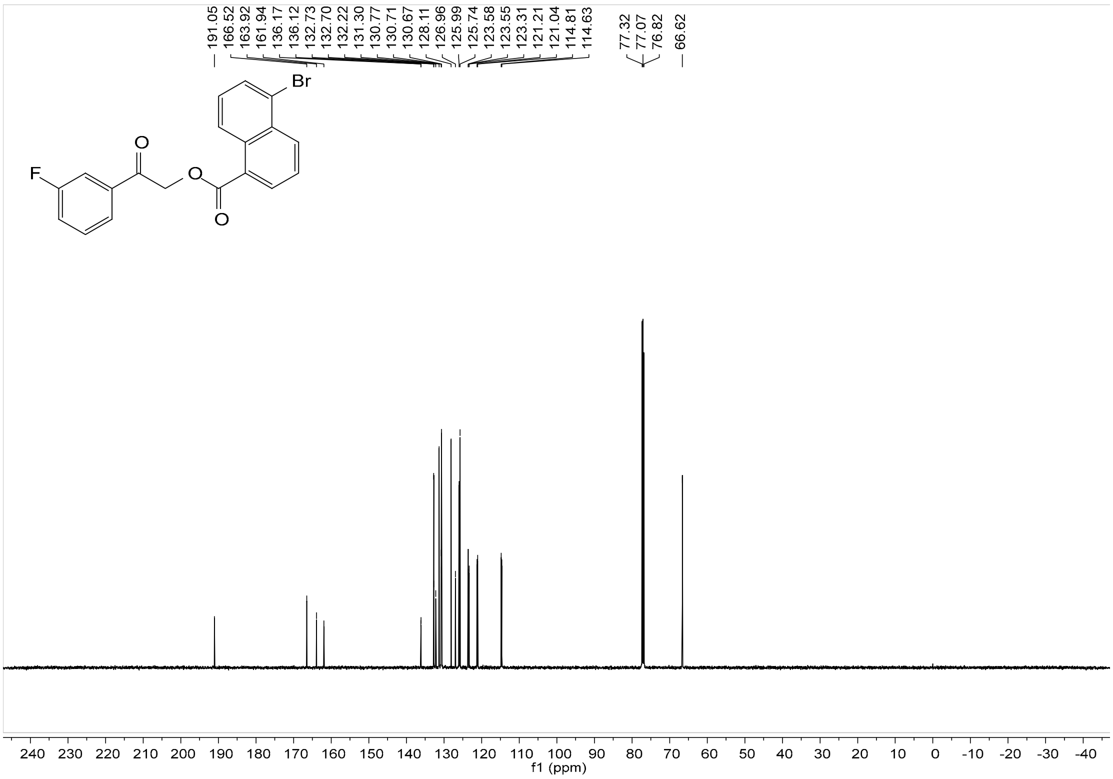

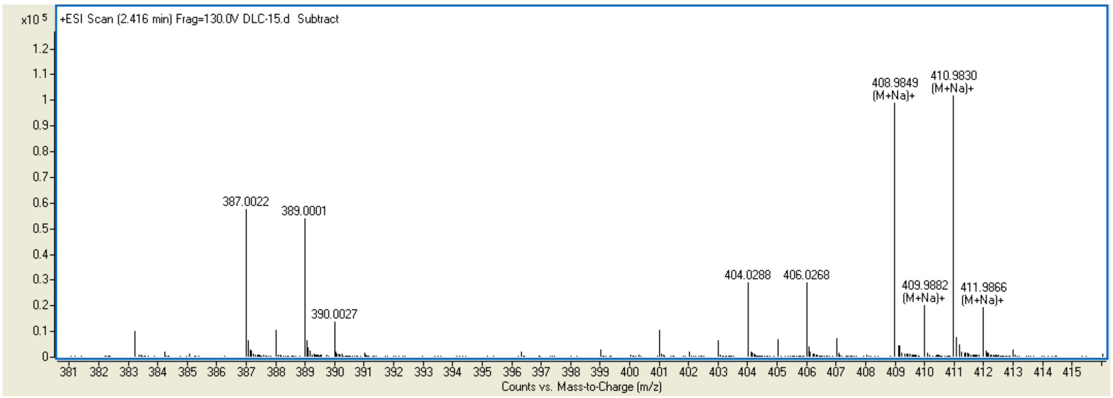

Figure S2. ^{1}H, ^{13}C and HRMS spectra of **C2**

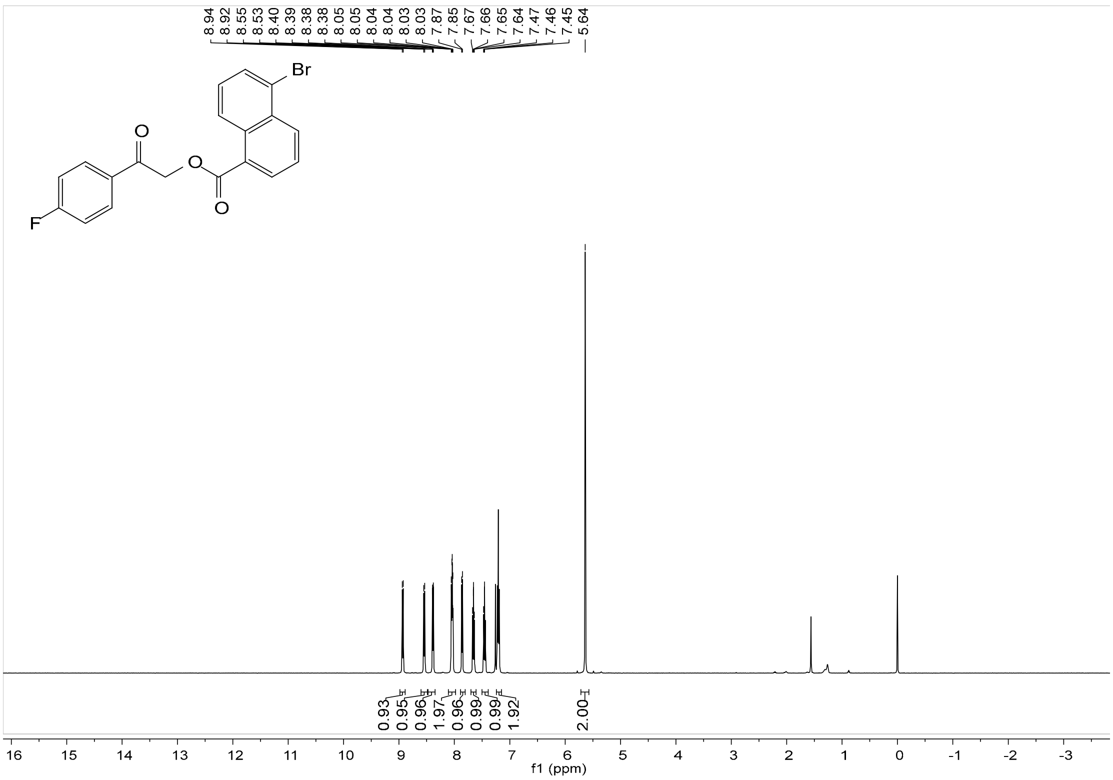
8.94
8.92
8.55
8.53
8.40
8.39
8.38
8.38
8.05
8.05
8.04
8.04
8.03
8.03
7.87
7.85
7.67
7.66
7.65
7.64
7.47
7.46
7.45
5.64
Br
O
O
O
F
0.93
0.95
0.96
1.97
0.96
0.99
0.99
1.92
2.00
f1 (ppm)

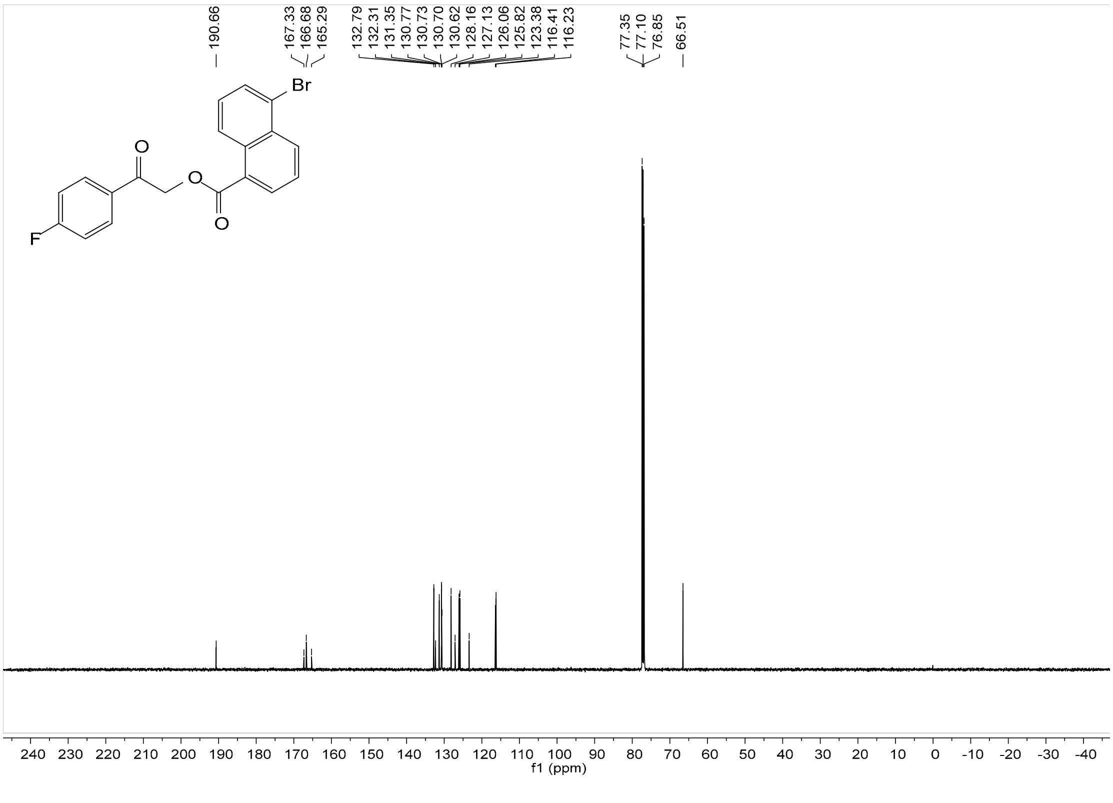
190.66
167.33
166.68
165.29
132.79
132.31
131.35
130.77
130.73
130.70
130.62
128.16
127.13
126.06
125.82
123.38
116.41
116.23
77.35
77.10
76.85
66.51
Br
O
O
O
F
f1 (ppm)

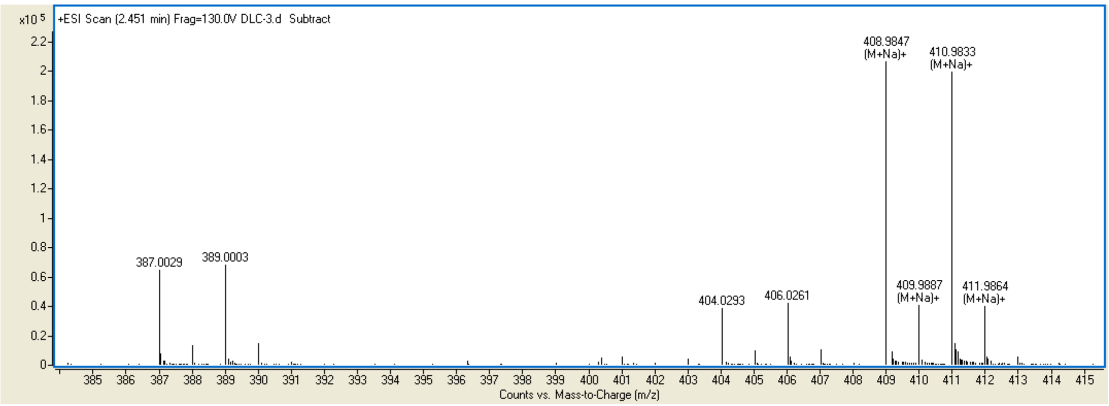

Figure S3. 1H, 13C and HRMS spectra of C3

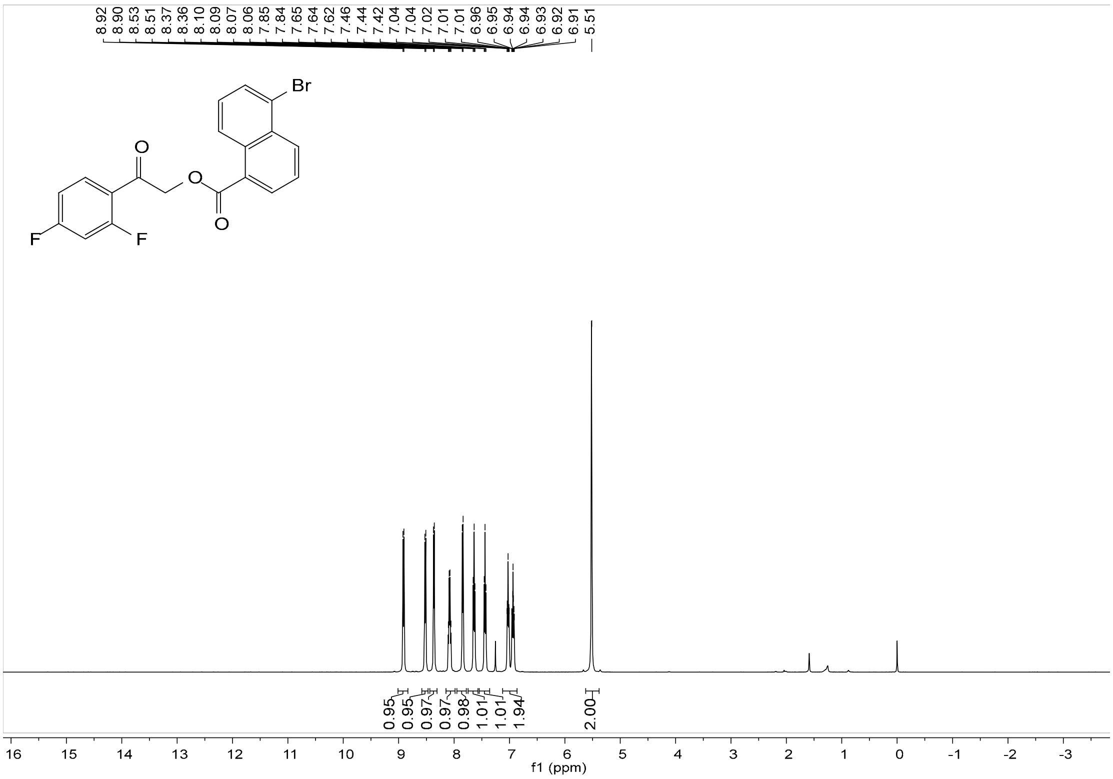

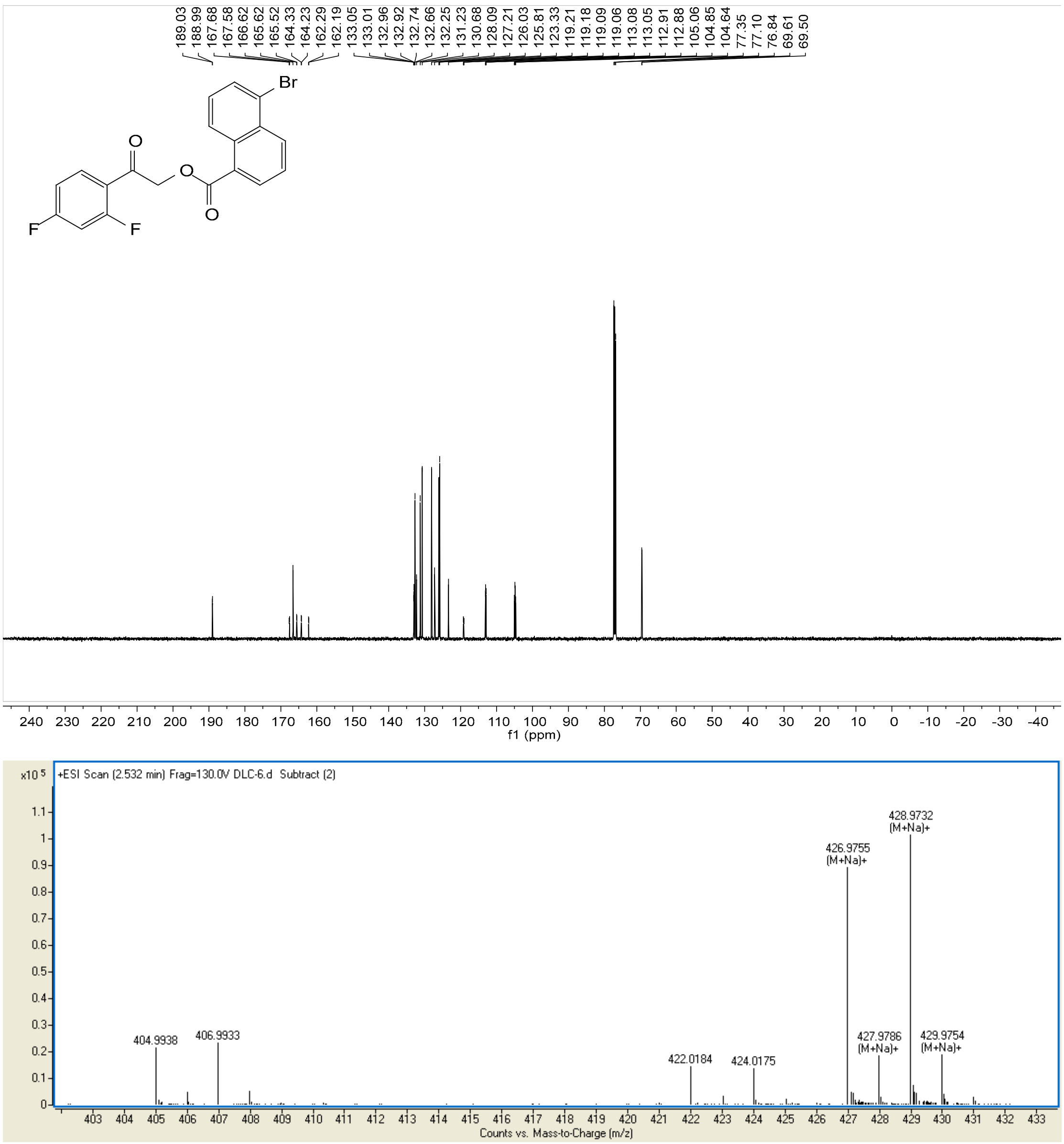

Figure S4. ^{1}H, ^{13}C and HRMS spectra of **C4**

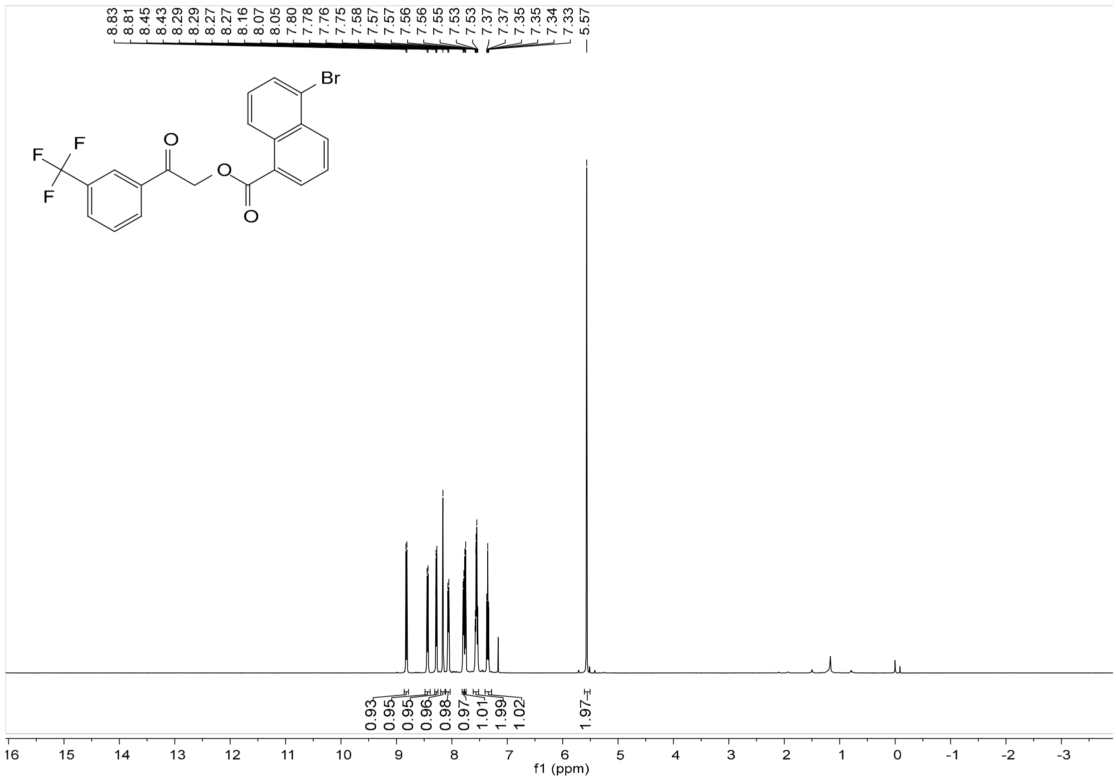

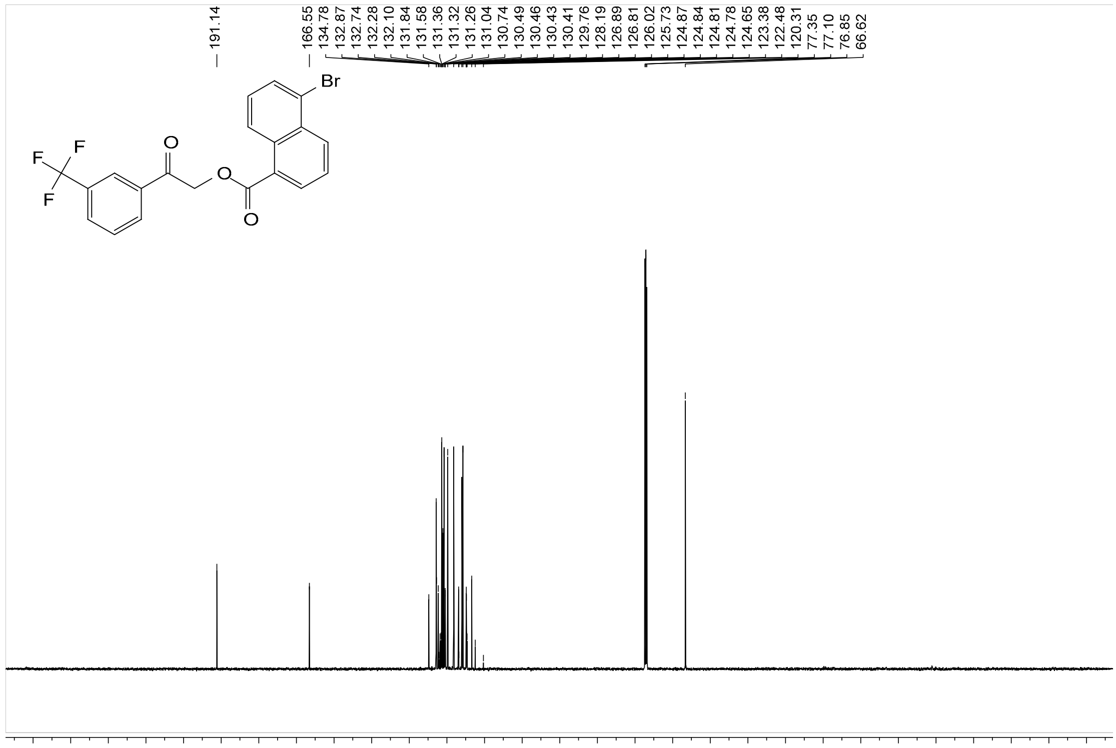

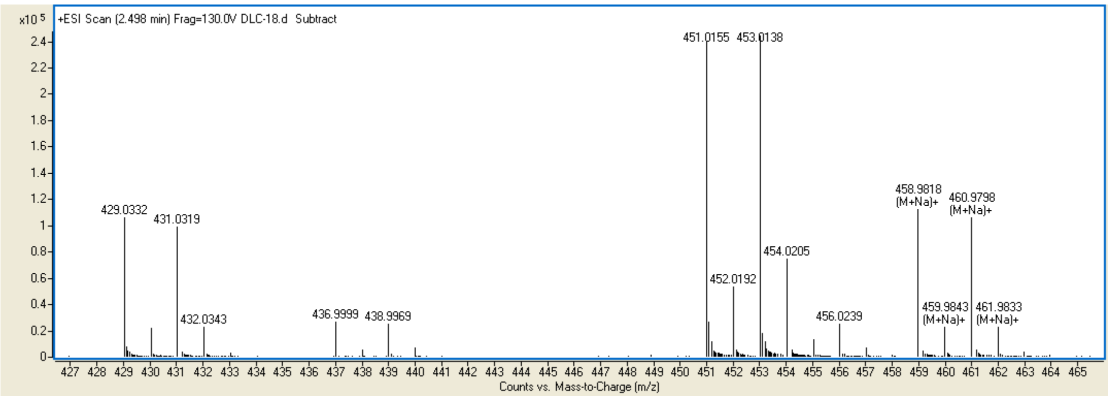

Figure S5. ^{1}H, ^{13}C and HRMS spectra of **C5**

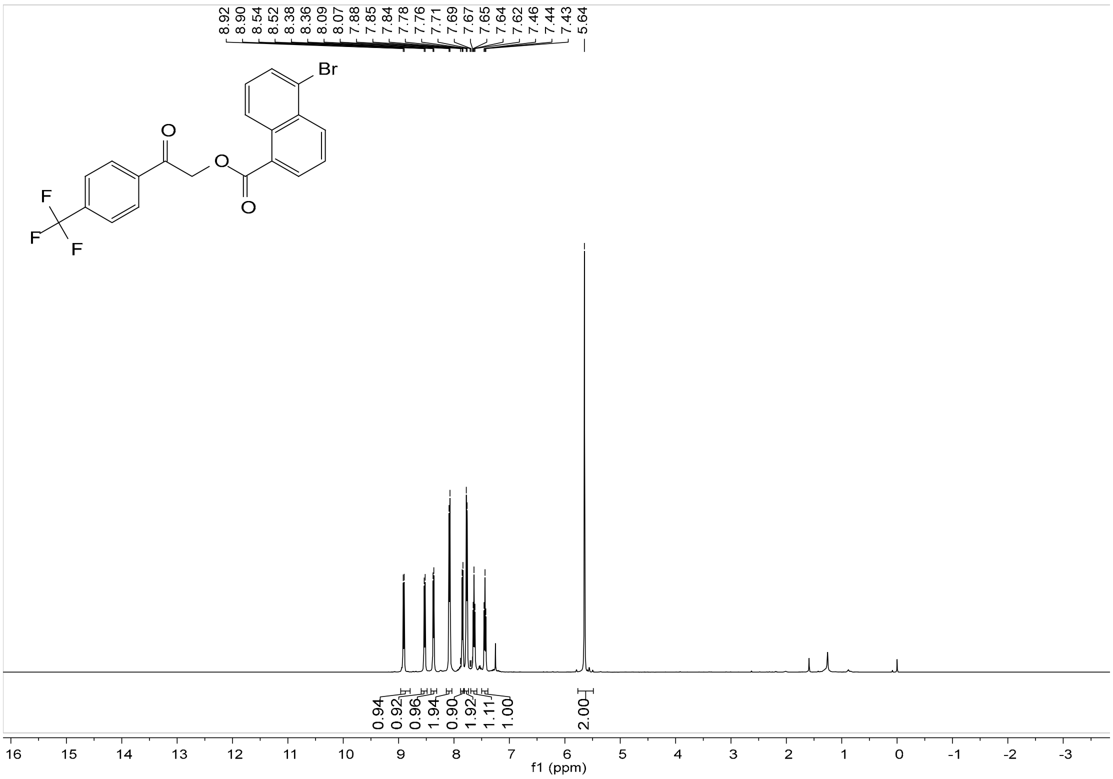

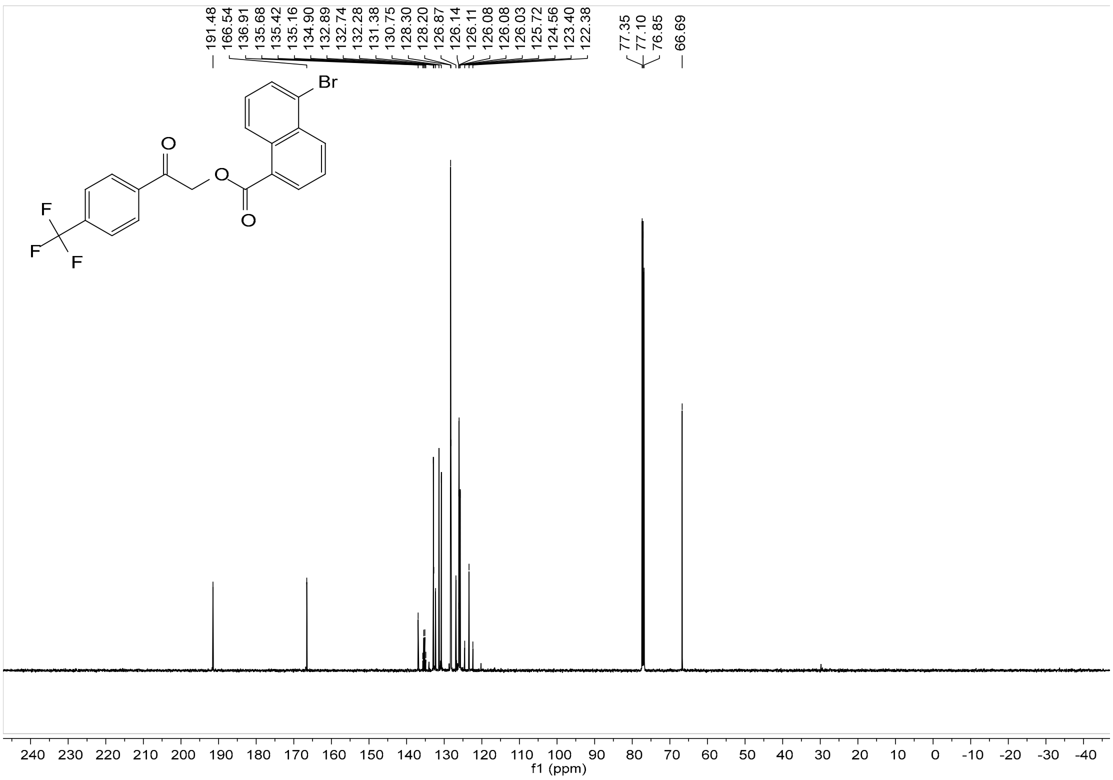

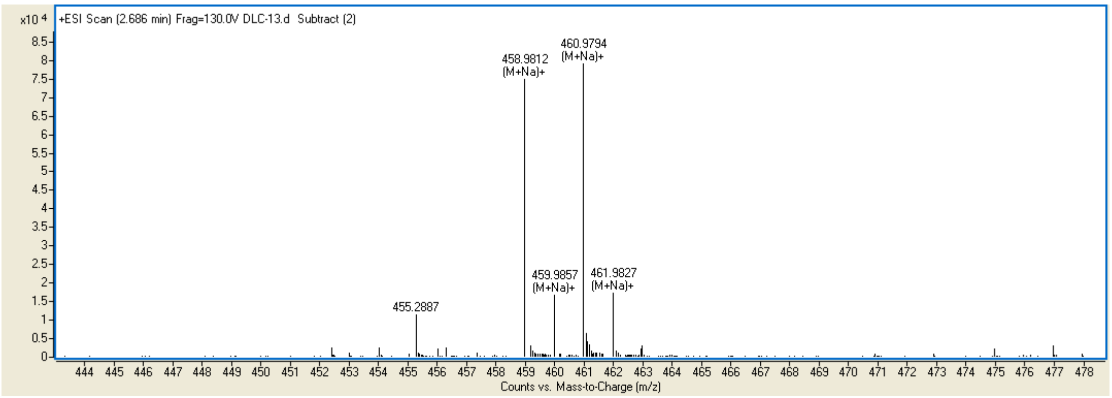

Figure S6. ^{1}H, ^{13}C and HRMS spectra of **C6**

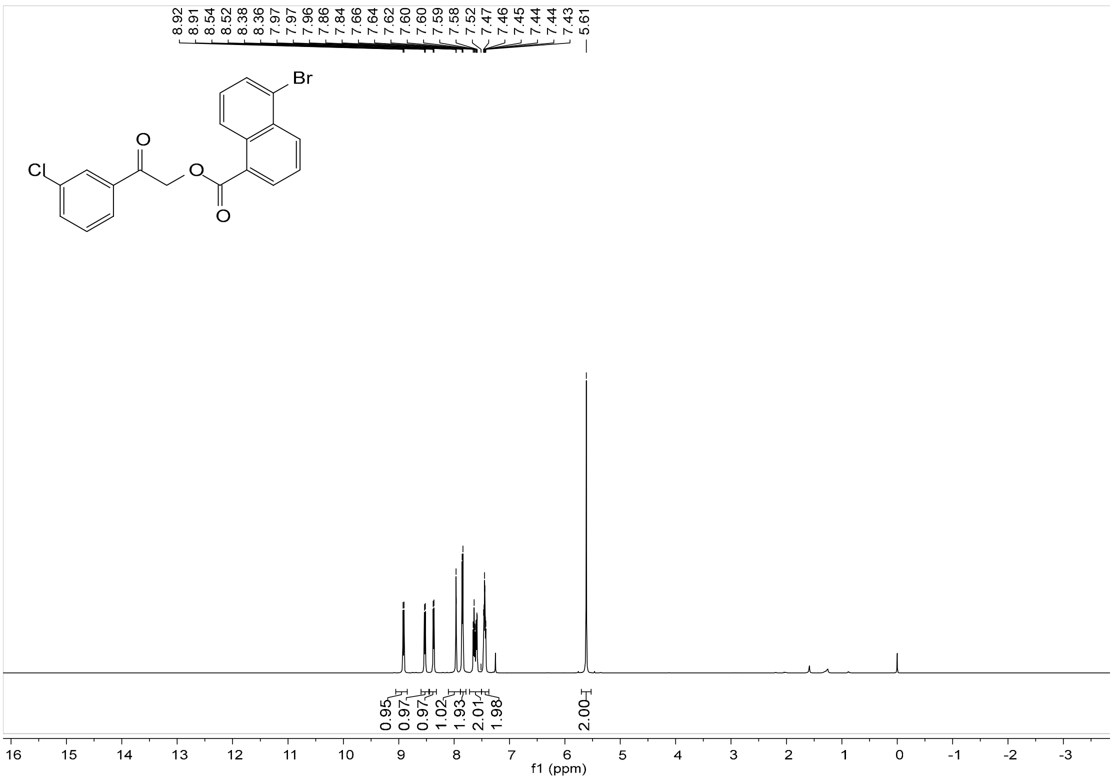
8.92
8.91
8.54
8.52
8.38
8.36
7.97
7.97
7.96
7.86
7.84
7.66
7.64
7.62
7.60
7.60
7.59
7.58
7.52
7.47
7.46
7.45
7.44
7.44
7.43
5.61
Br
O
Cl
O
O
0.95
0.97
0.97
1.02
1.93
2.01
1.98
2.00
f1 (ppm)

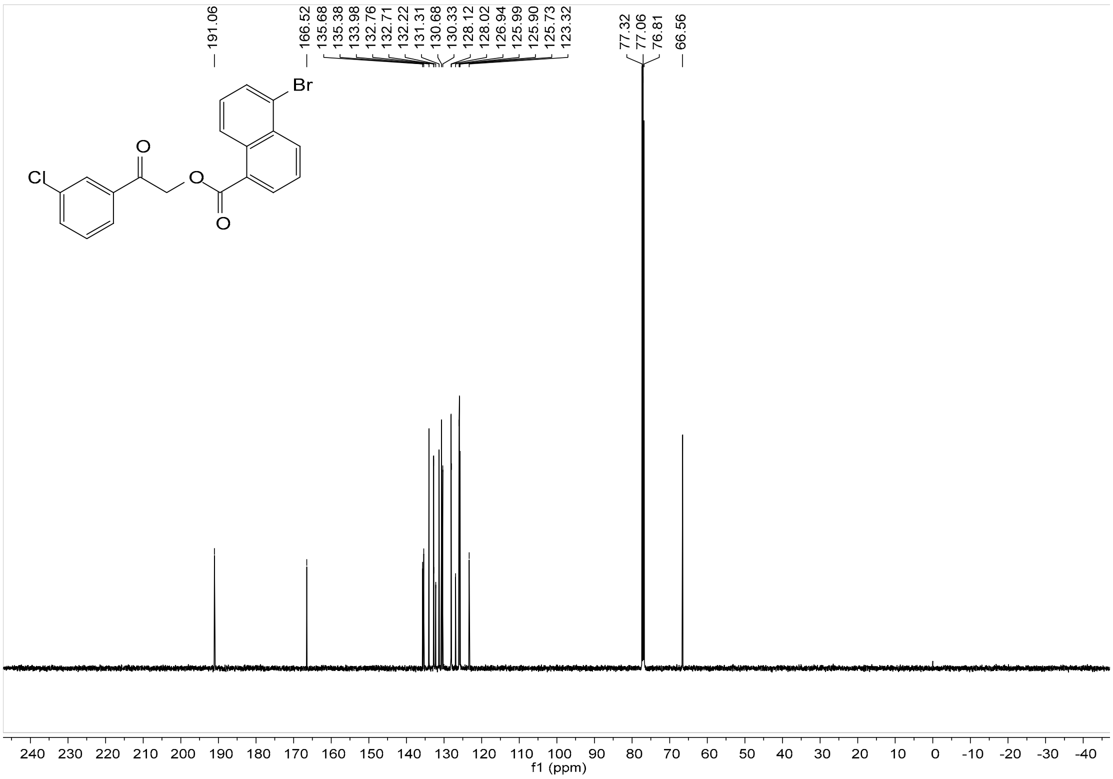
191.06
166.52
135.68
135.38
133.98
132.76
132.71
132.22
131.31
130.68
130.33
128.12
128.02
126.94
125.99
125.90
125.73
123.32
77.32
77.06
76.81
66.56
Br
O
Cl
O
O
f1 (ppm)

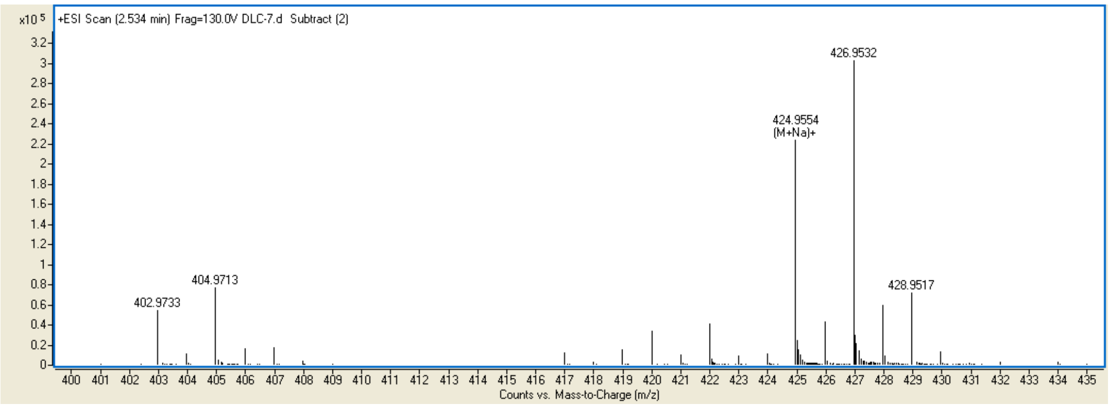

Figure S7. ^{1}H, ^{13}C and HRMS spectra of **C7**

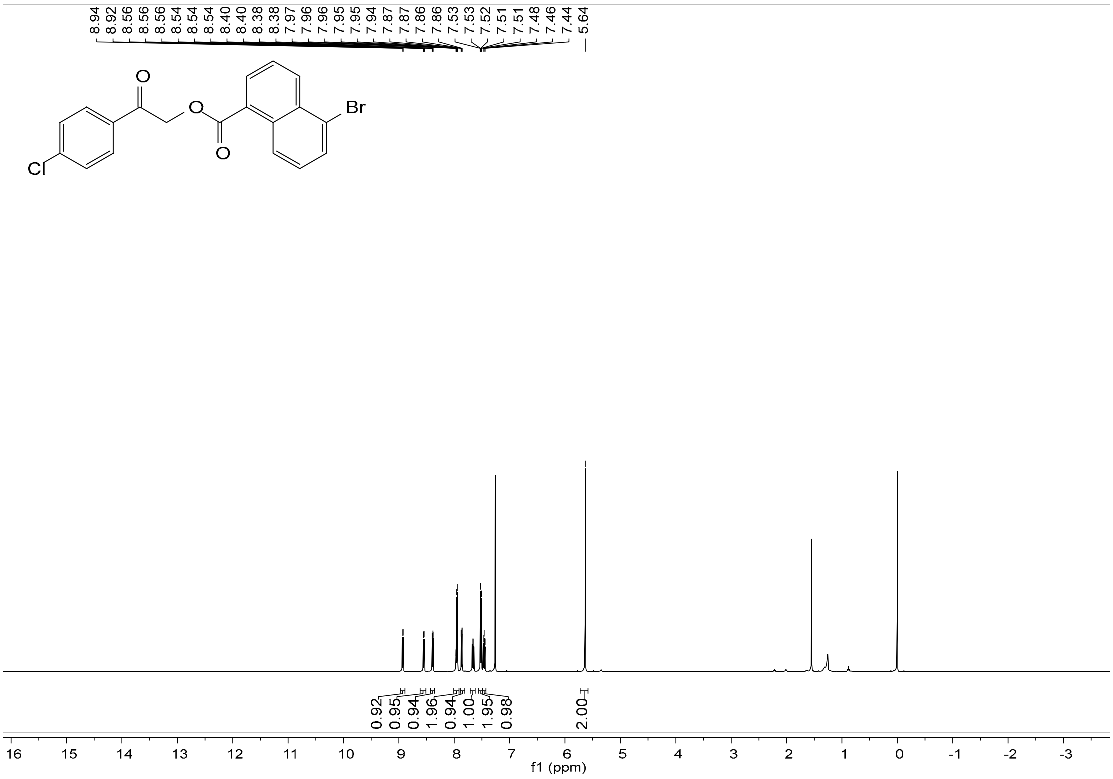

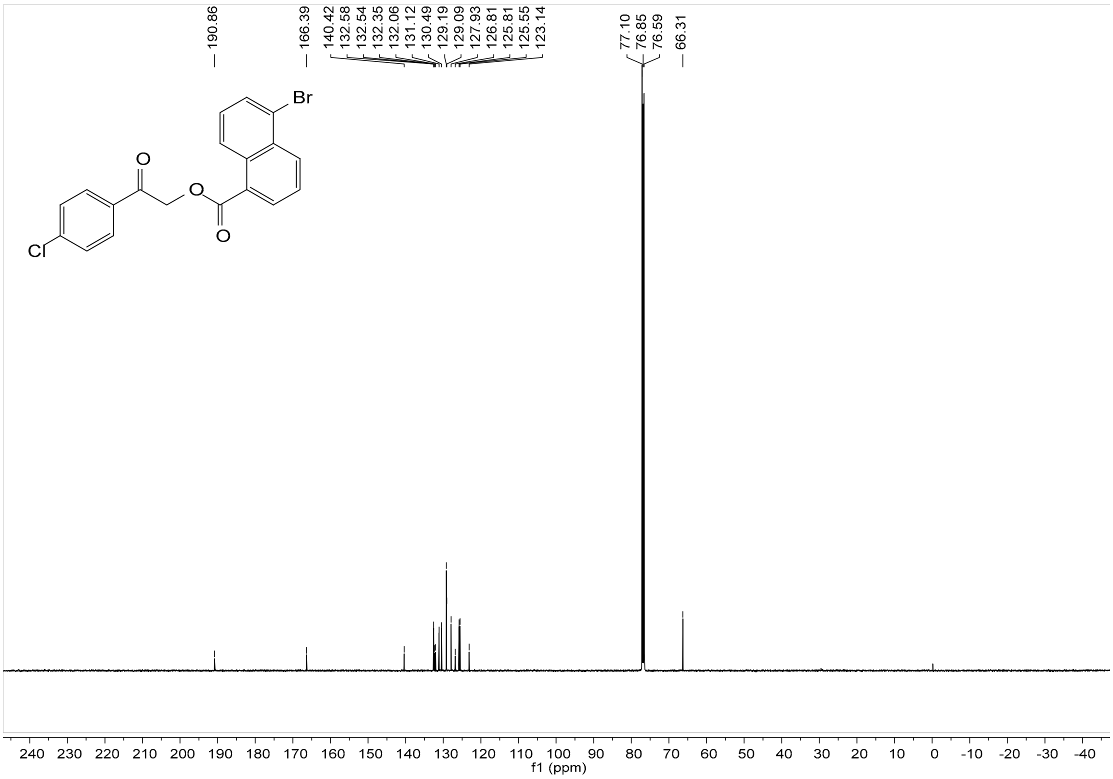

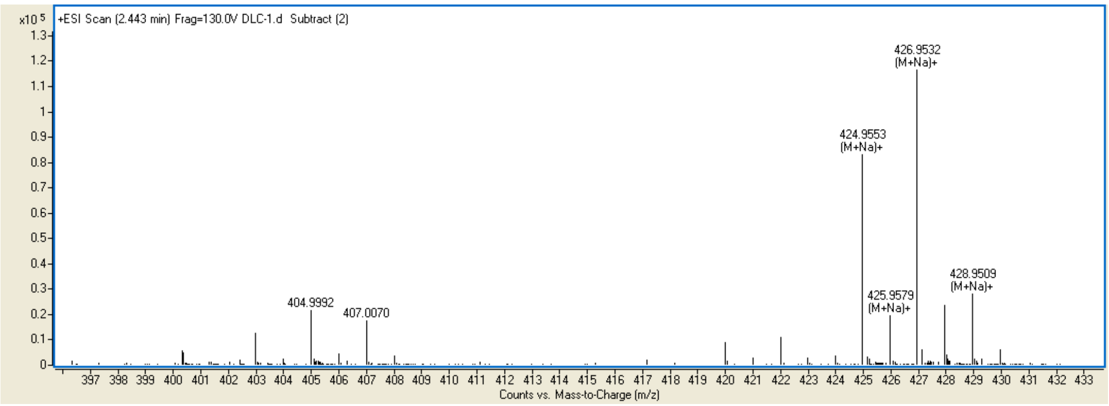

Figure S8. ^{1}H, ^{13}C and HRMS spectra of **C8**

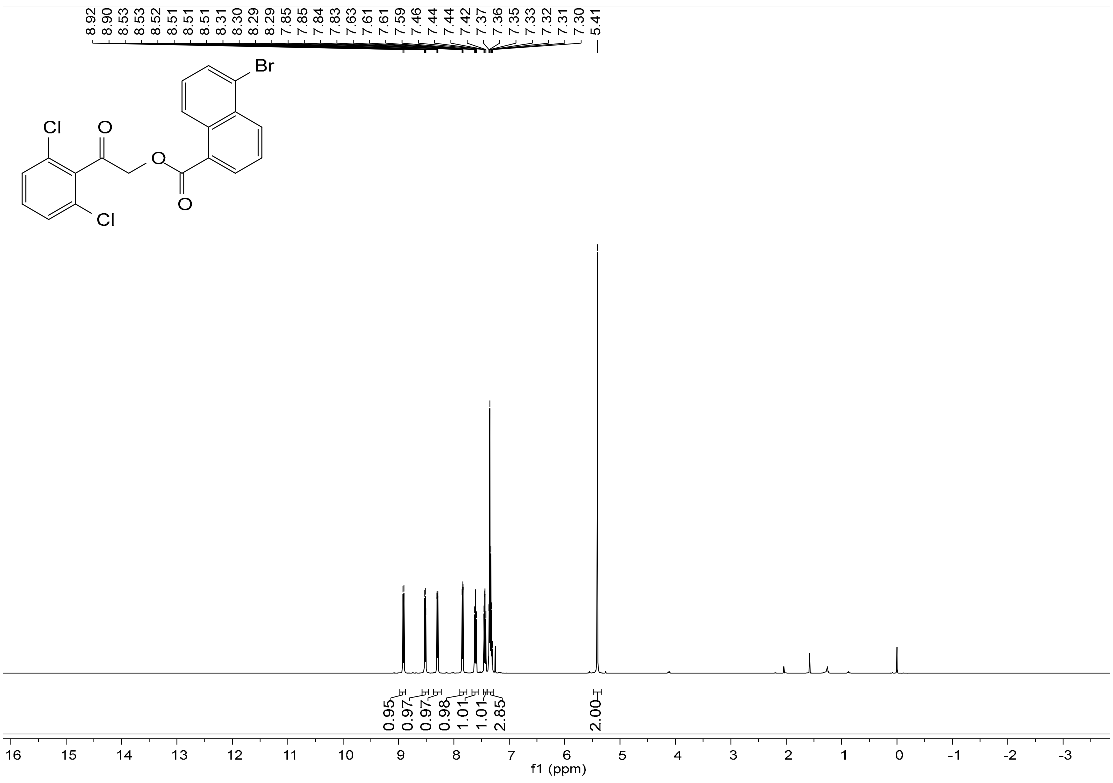
8.92
8.90
8.53
8.52
8.51
8.51
8.51
8.31
8.30
8.29
8.29
7.85
7.84
7.83
7.63
7.61
7.61
7.59
7.46
7.44
7.44
7.42
7.37
7.36
7.35
7.33
7.32
7.31
7.30
5.41
Br
Cl
O
O
O
Cl
0.95
0.97
0.97
0.98
1.01
1.01
2.85
2.00
16 15 14 13 12 11 10 9 8 7 6 5 4 3 2 1 0 -1 -2 -3
f1 (ppm)

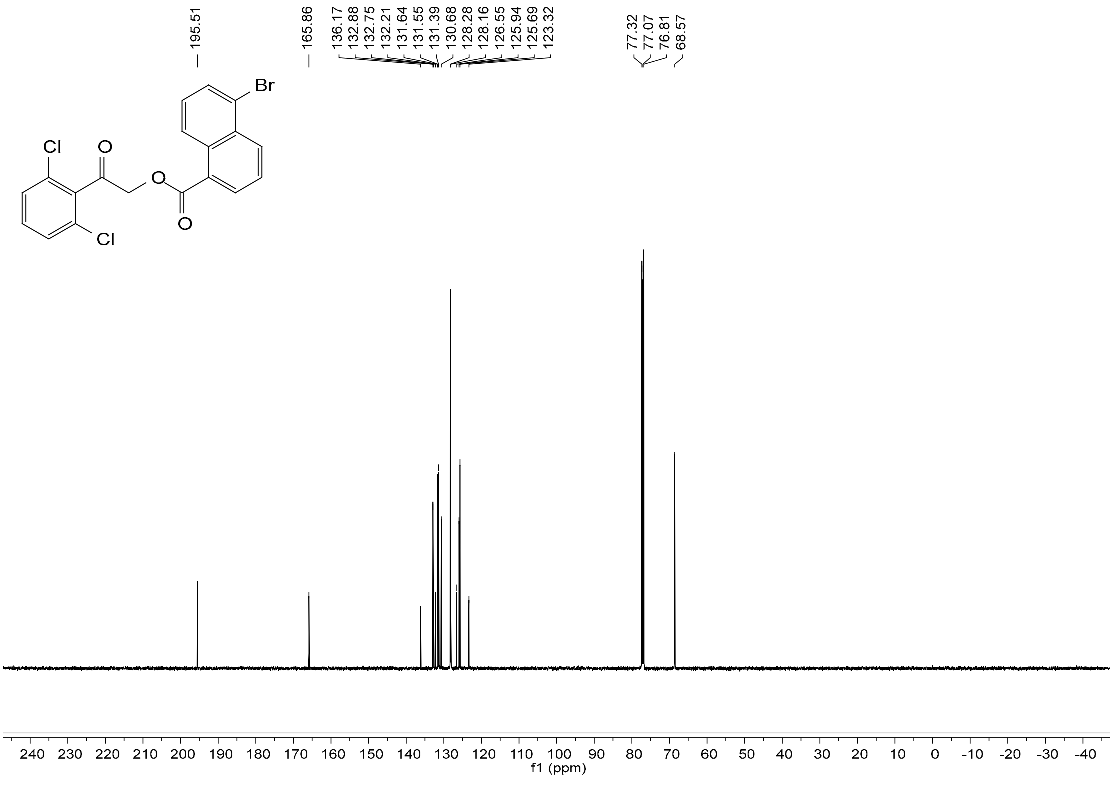
195.51
165.86
136.17
132.88
132.75
132.21
131.64
131.55
131.39
130.68
128.28
128.16
126.55
125.94
125.69
123.32
77.32
77.07
76.81
68.57
Br
Cl
O
O
O
Cl
240 230 220 210 200 190 180 170 160 150 140 130 120 110 100 90 80 70 60 50 40 30 20 10 0 -10 -20 -30 -40
f1 (ppm)

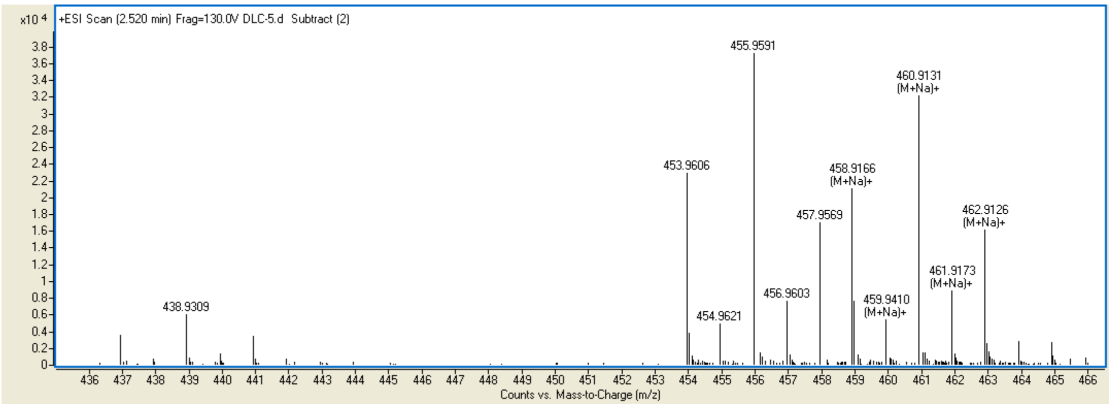

Figure S9. ^{1}H, ^{13}C and HRMS spectra of **C9**

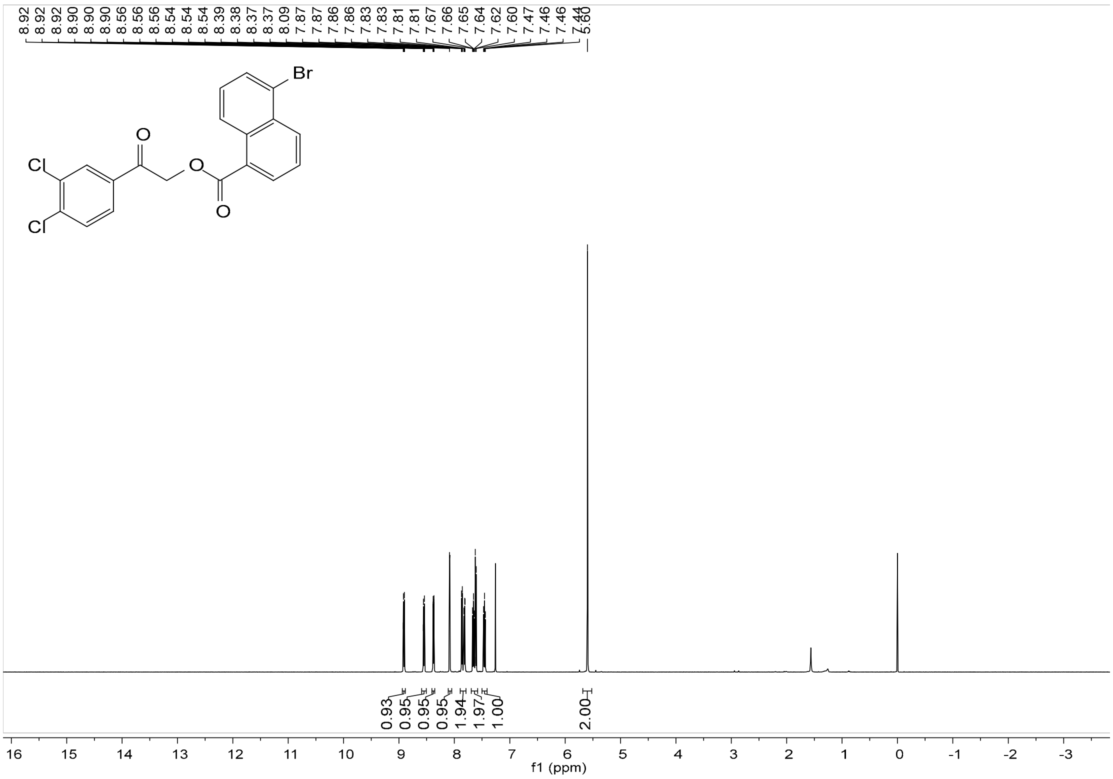

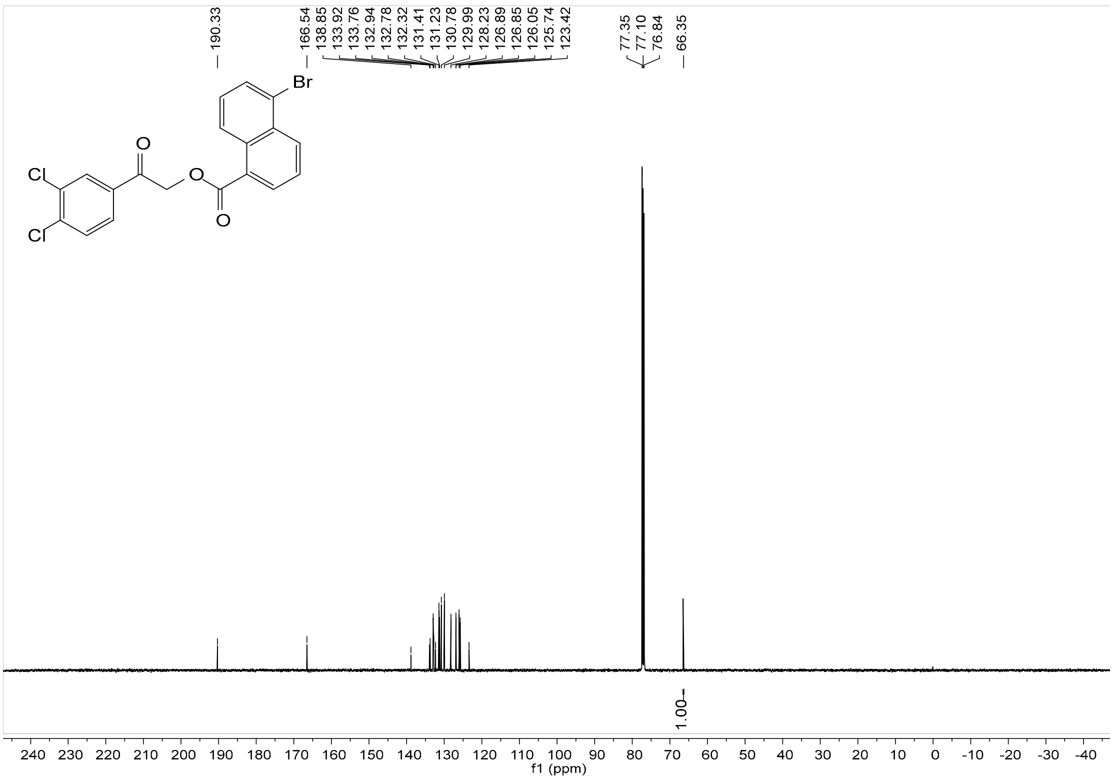

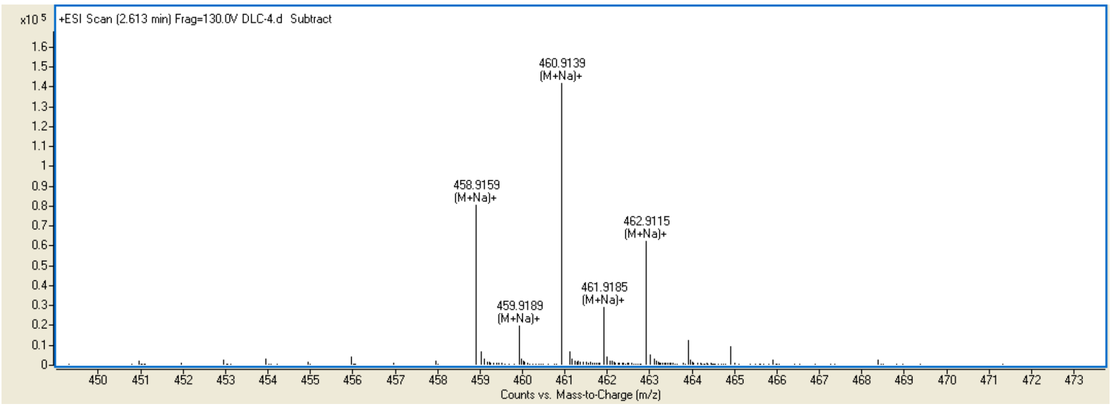

Figure S10. ^{1}H, ^{13}C and HRMS spectra of **C10**

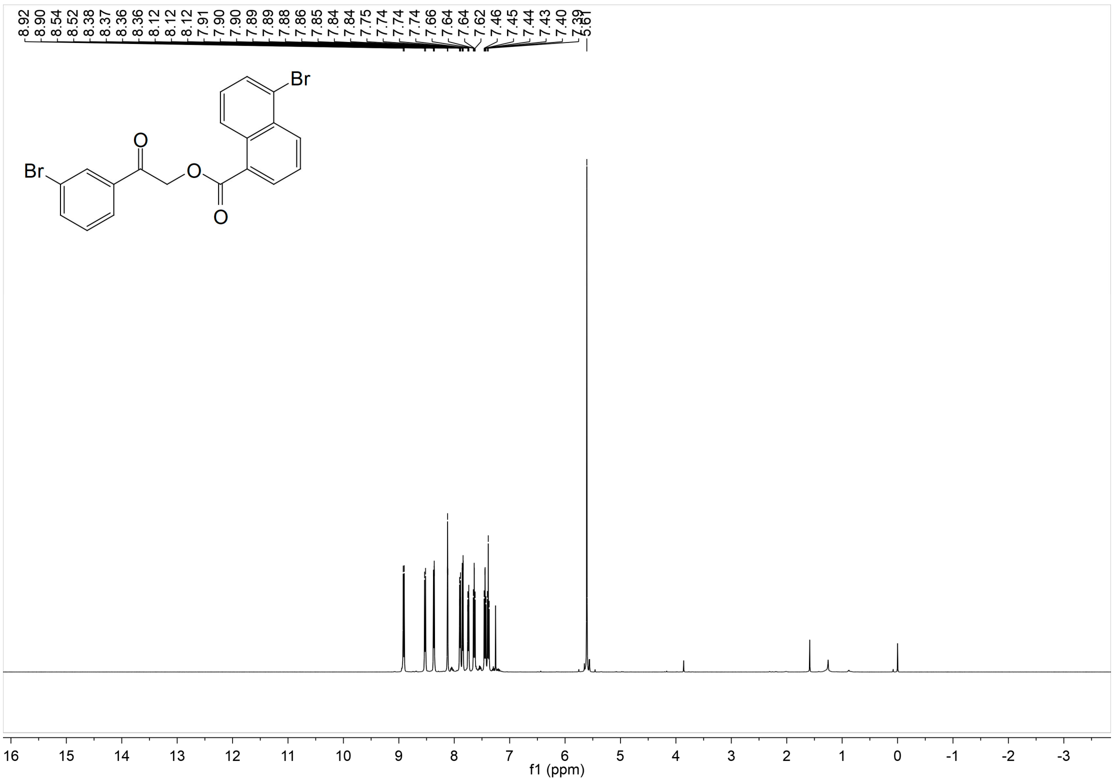
8.92
8.90
8.54
8.52
8.38
8.37
8.36
8.36
8.12
8.12
8.12
7.91
7.90
7.90
7.89
7.88
7.86
7.85
7.84
7.84
7.75
7.74
7.74
7.74
7.66
7.64
7.62
7.46
7.45
7.44
7.43
7.40
7.39
5.61
Br
O
Br
O
O
16 15 14 13 12 11 10 9 8 7 6 5 4 3 2 1 0 -1 -2 -3
f1 (ppm)

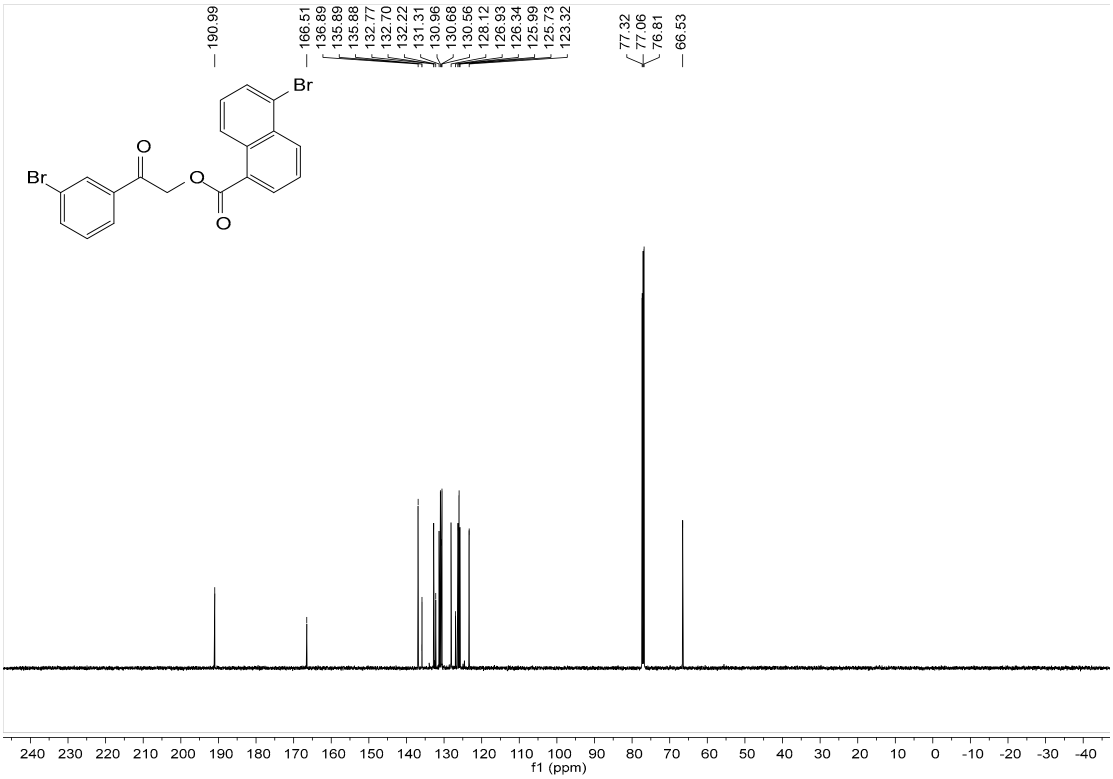
190.99
166.51
136.89
135.89
135.88
132.77
132.70
132.22
131.31
130.96
130.68
130.56
128.12
126.93
126.34
125.99
125.73
123.32
77.32
77.06
76.81
66.53
Br
O
Br
O
O
240 230 220 210 200 190 180 170 160 150 140 130 120 110 100 90 80 70 60 50 40 30 20 10 0 -10 -20 -30 -40
f1 (ppm)

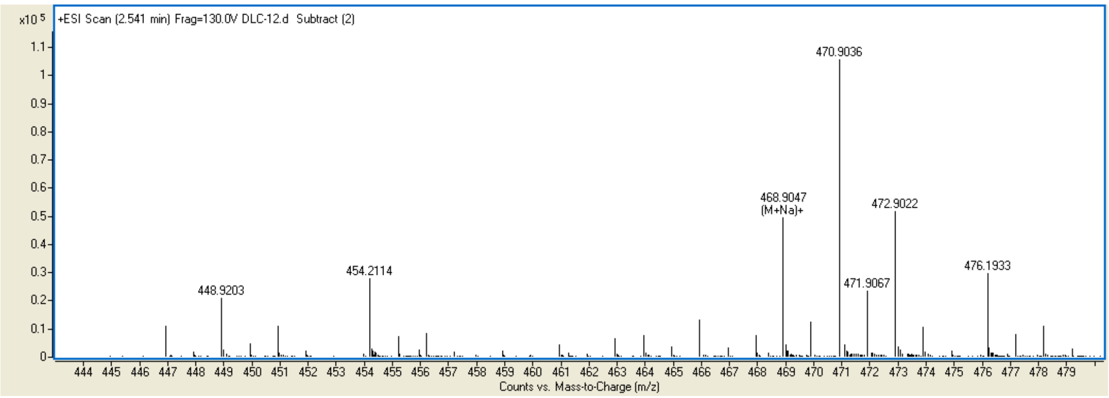

Figure S11. ^{1}H, ^{13}C and HRMS spectra of **C11**

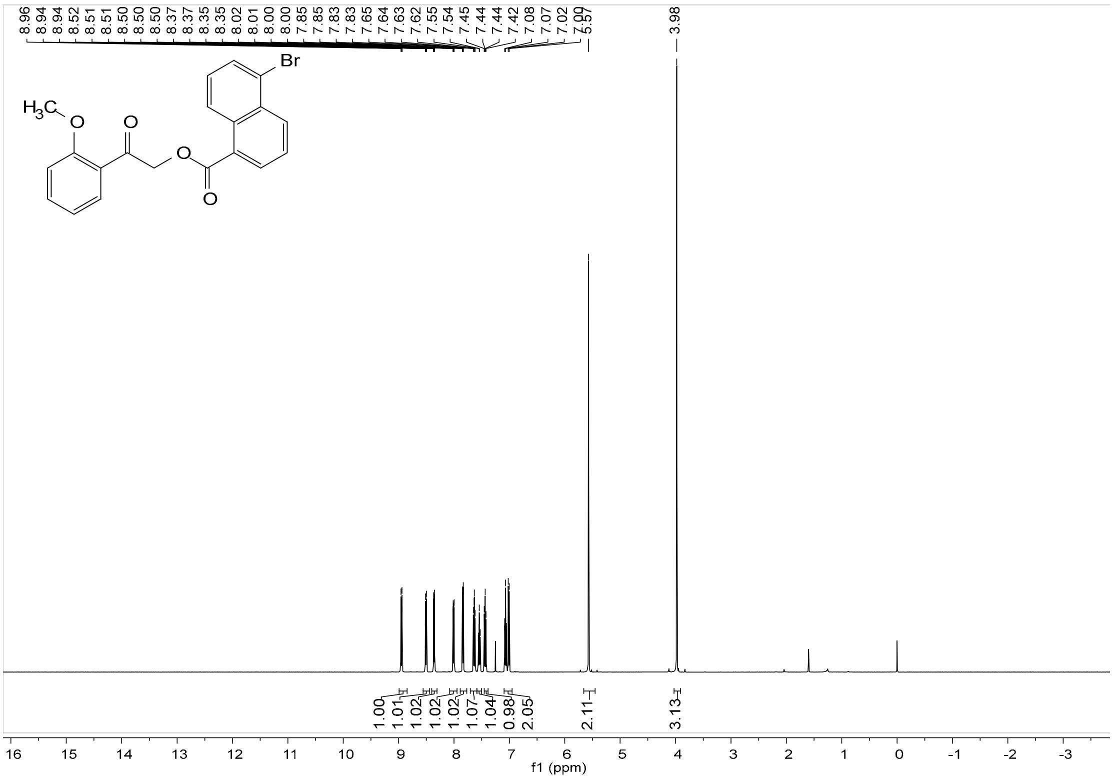

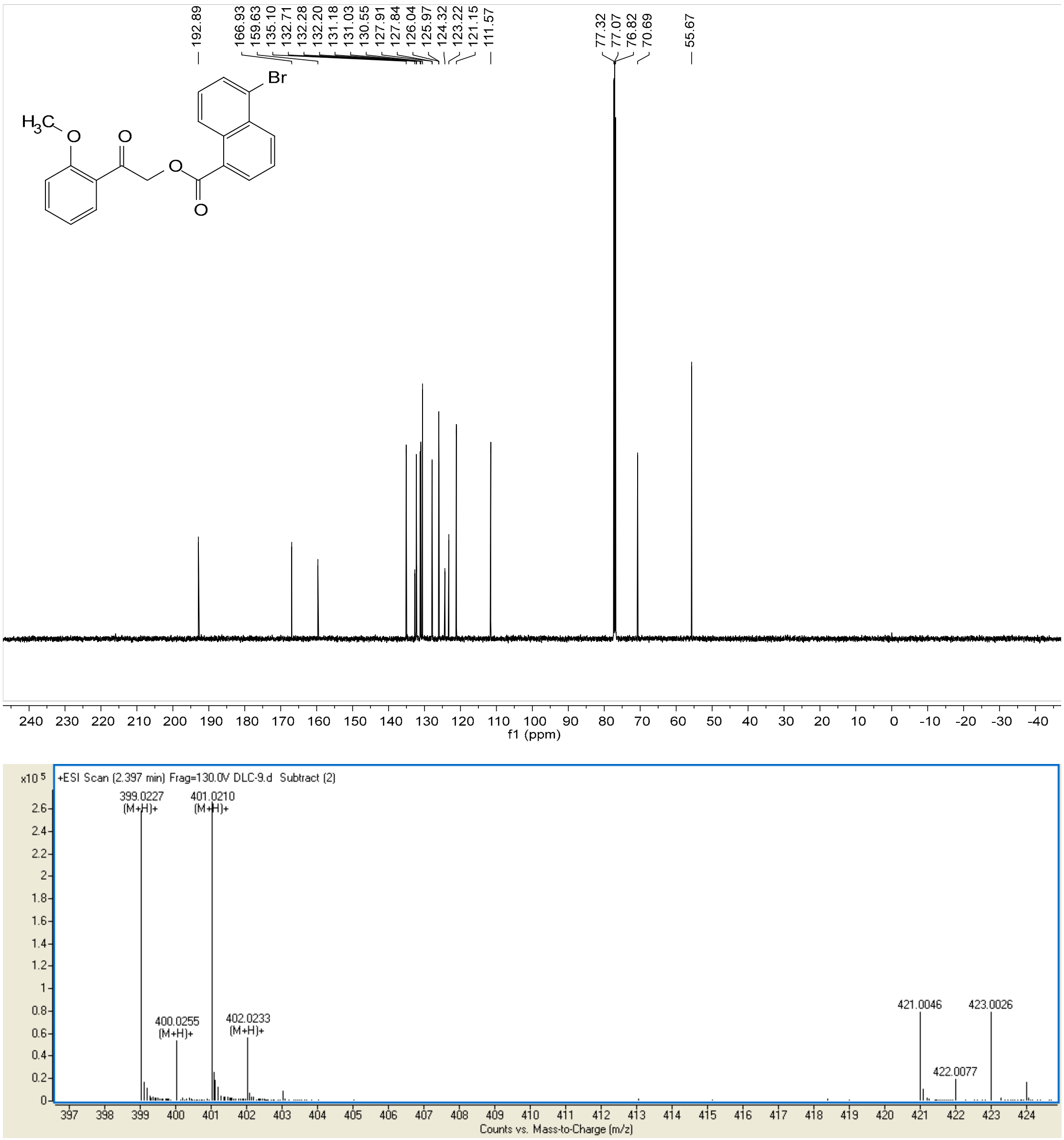

Figure S12. ^{1}H, ^{13}C and HRMS spectra of **C12**

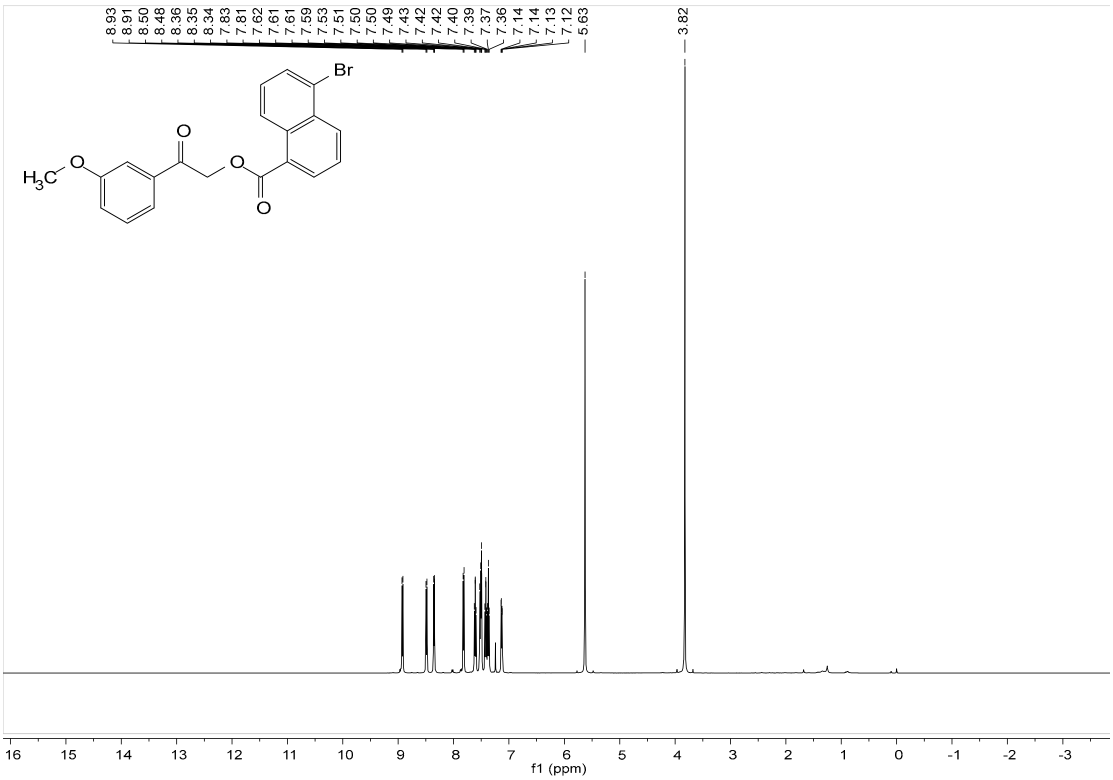
8.93
8.91
8.50
8.48
8.36
8.35
8.34
7.83
7.81
7.62
7.61
7.61
7.59
7.53
7.51
7.50
7.50
7.49
7.43
7.42
7.40
7.39
7.37
7.36
7.14
7.14
7.13
7.12
5.63
3.82
Br
O
H3C
O
O
O
f1 (ppm)

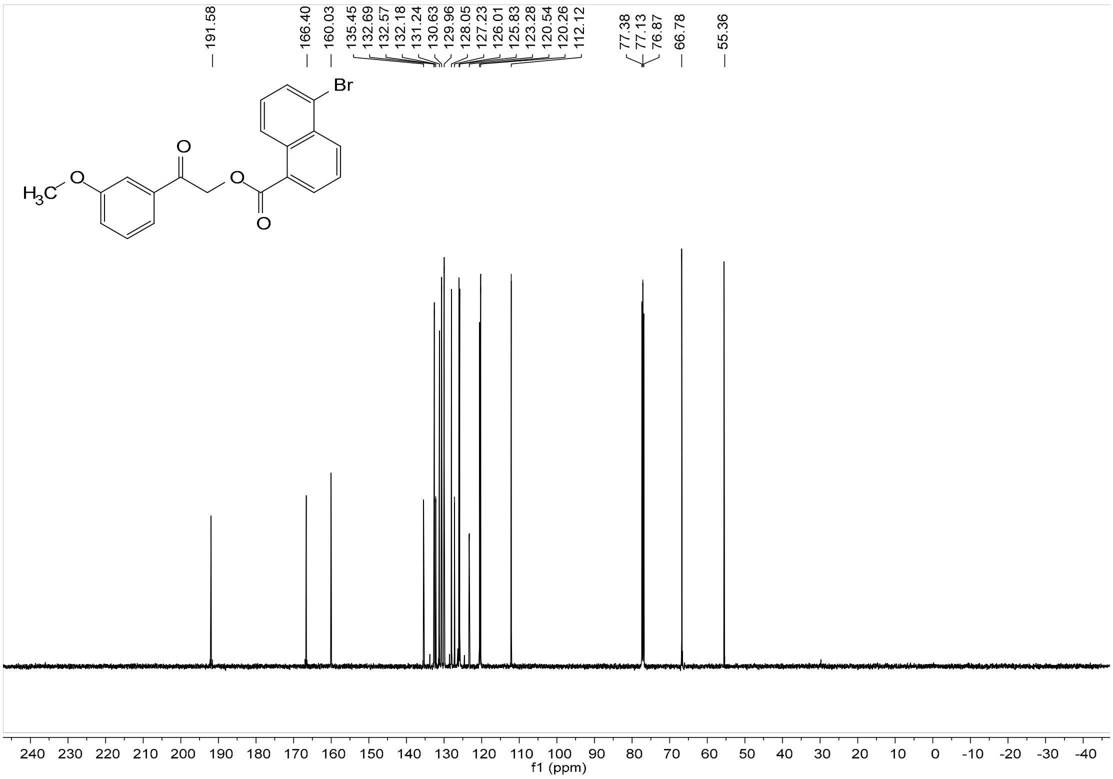
191.58
166.40
160.03
135.45
132.69
132.57
132.18
131.24
130.63
129.96
128.05
127.23
126.01
125.83
123.28
120.54
120.26
112.12
77.38
77.13
76.87
66.78
55.36
Br
O
H3C
O
O
O
f1 (ppm)

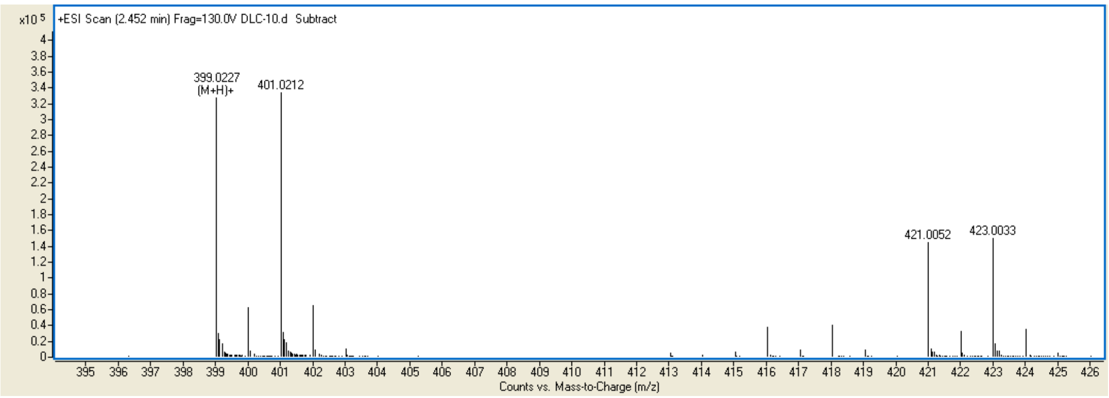

Figure S13. ^{1}H, ^{13}C and HRMS spectra of **C13**

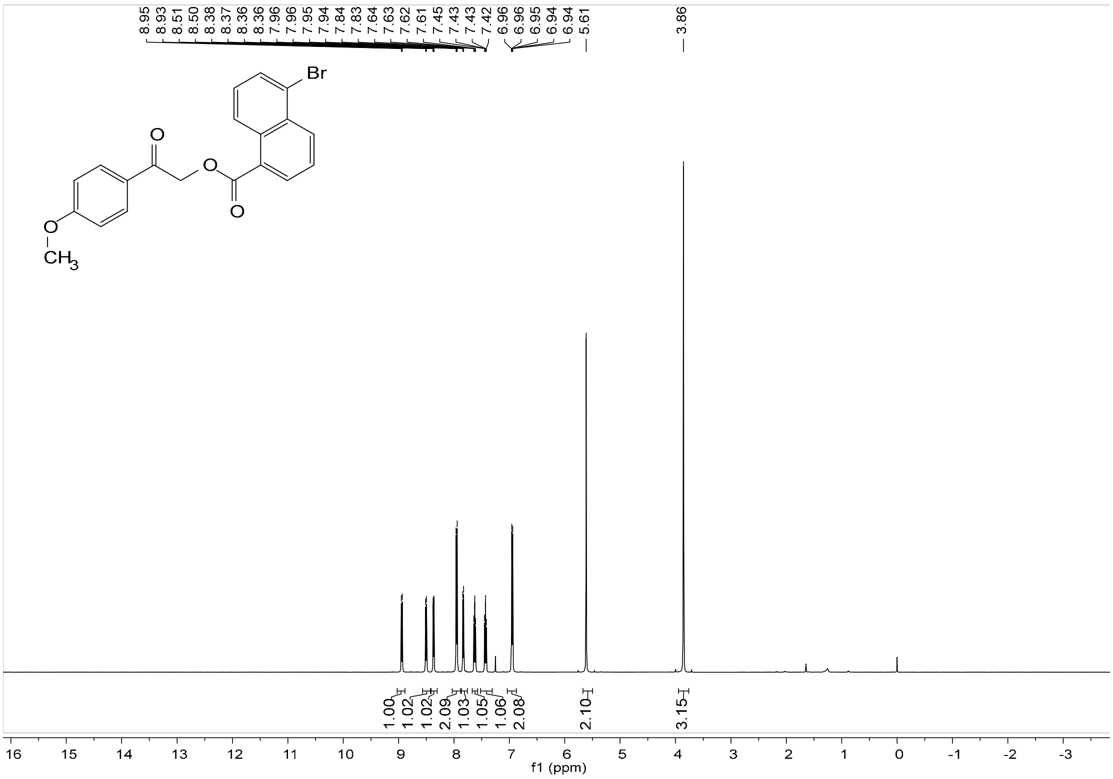

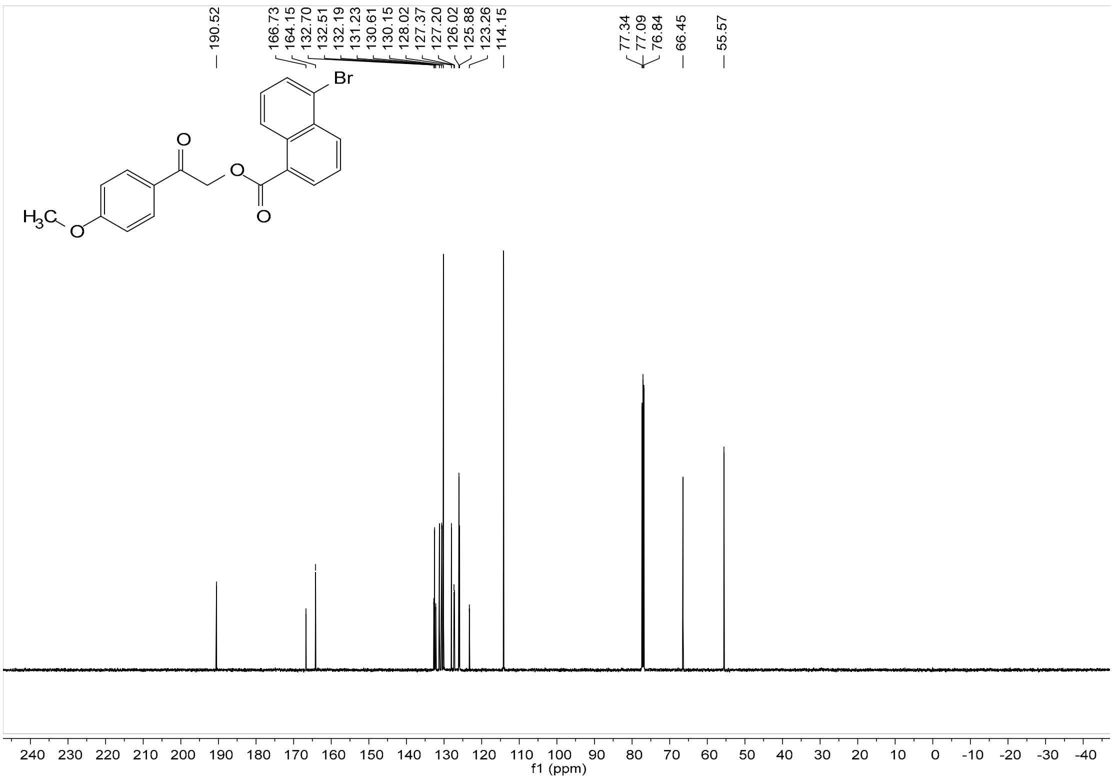

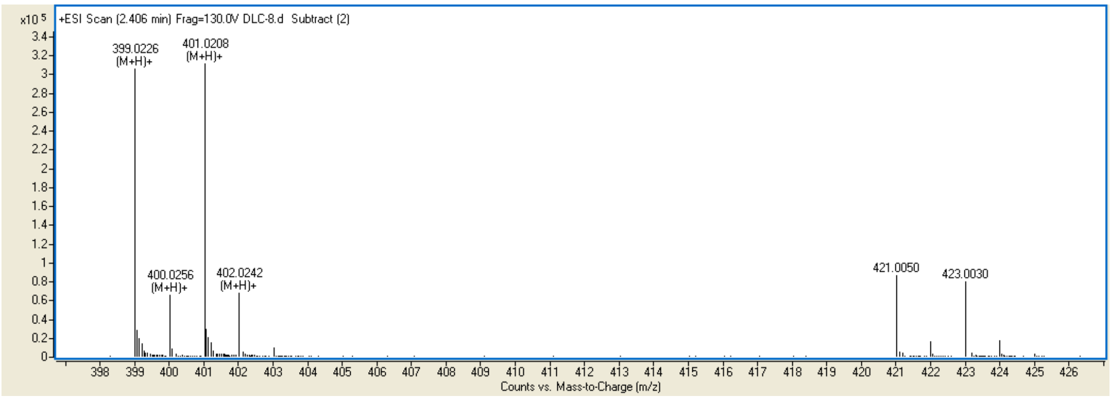

Figure S14. ^{1}H, ^{13}C and HRMS spectra of **C14**

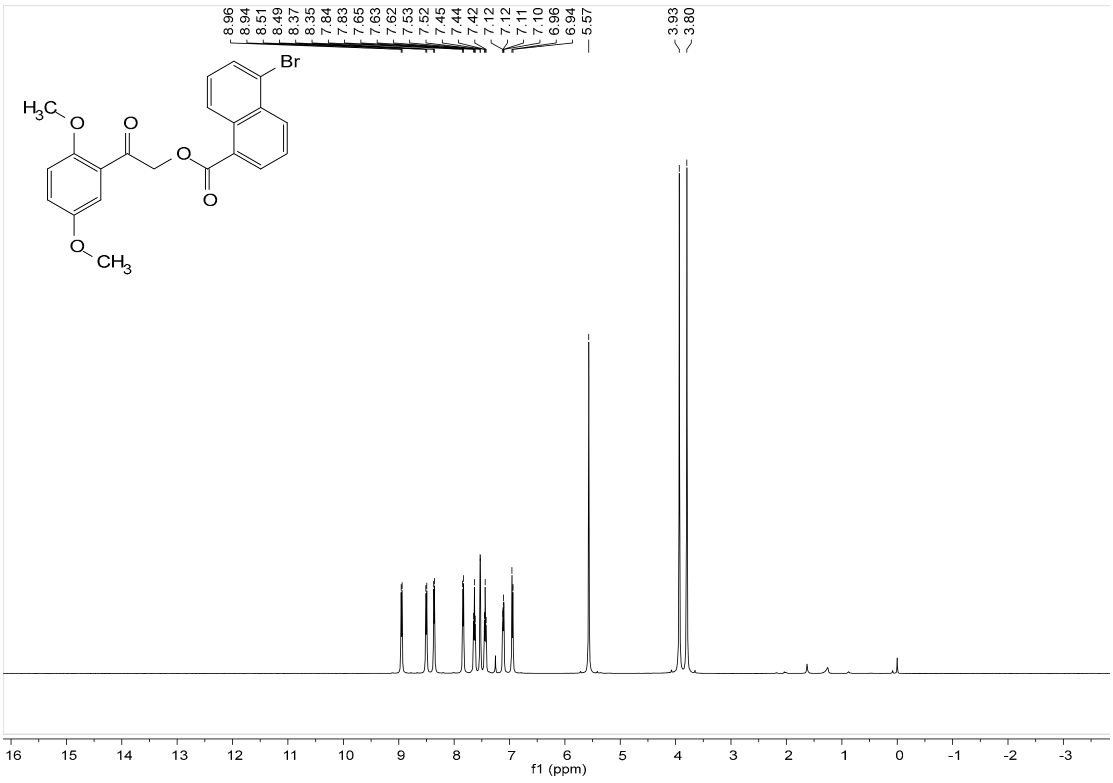
8.96 8.94 8.51 8.49 8.37 8.35 7.84 7.83 7.65 7.63 7.62 7.53 7.52 7.45 7.44 7.42 7.12 7.12 7.11 7.10 6.96 6.94 5.57
3.93 3.80
Br
H3C
O
O
O
O
O
CH3
16 15 14 13 12 11 10 9 8 7 6 5 4 3 2 1 0 -1 -2 -3
f1 (ppm)

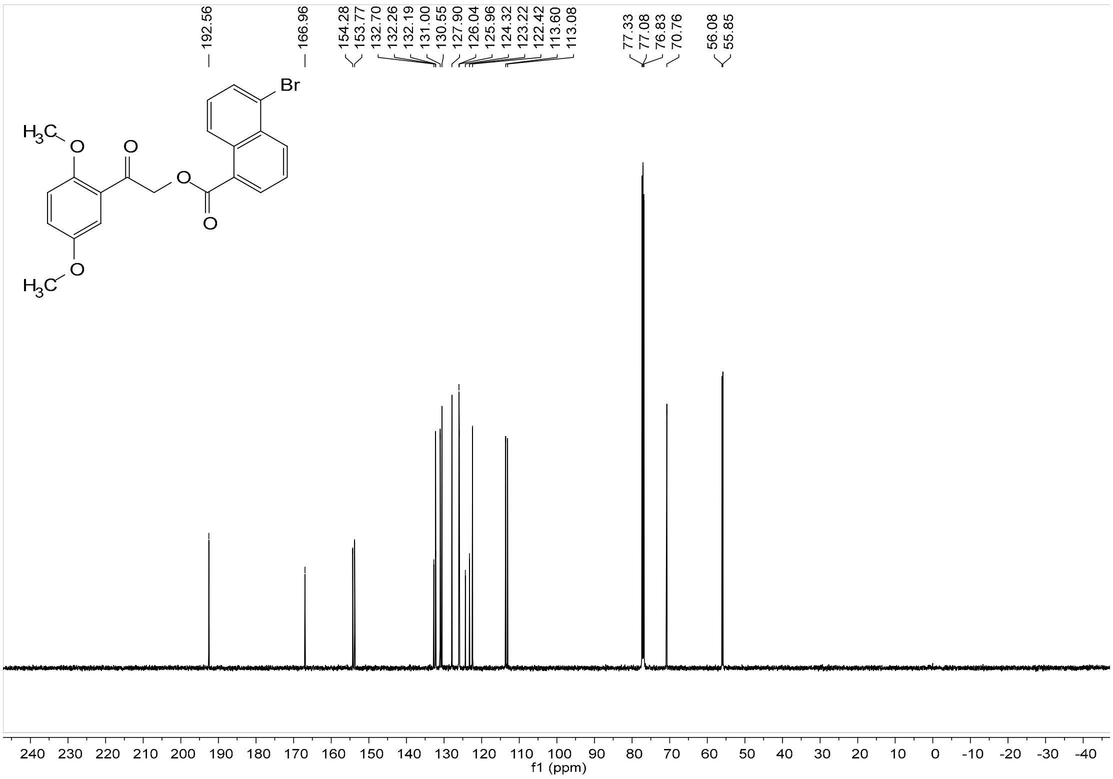
192.56
166.96
154.28 153.77 132.70 132.26 132.19 131.00 130.55 127.90 126.04 125.96 124.32 123.22 122.42 113.60 113.08
77.33 77.08 76.83 70.76
56.08 55.85
Br
H3C
O
O
O
O
O
H3C
240 230 220 210 200 190 180 170 160 150 140 130 120 110 100 90 80 70 60 50 40 30 20 10 0 -10 -20 -30 -40
f1 (ppm)

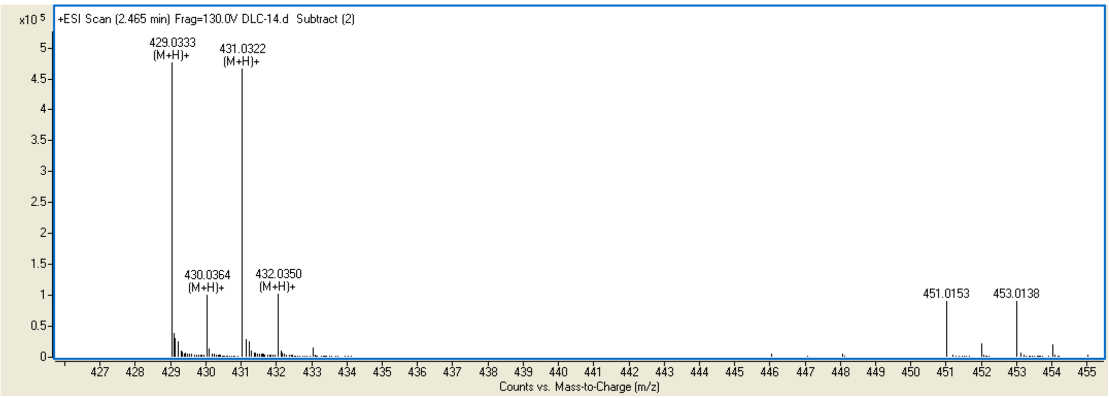

Figure S15. ^{1}H, ^{13}C and HRMS spectra of **C15**

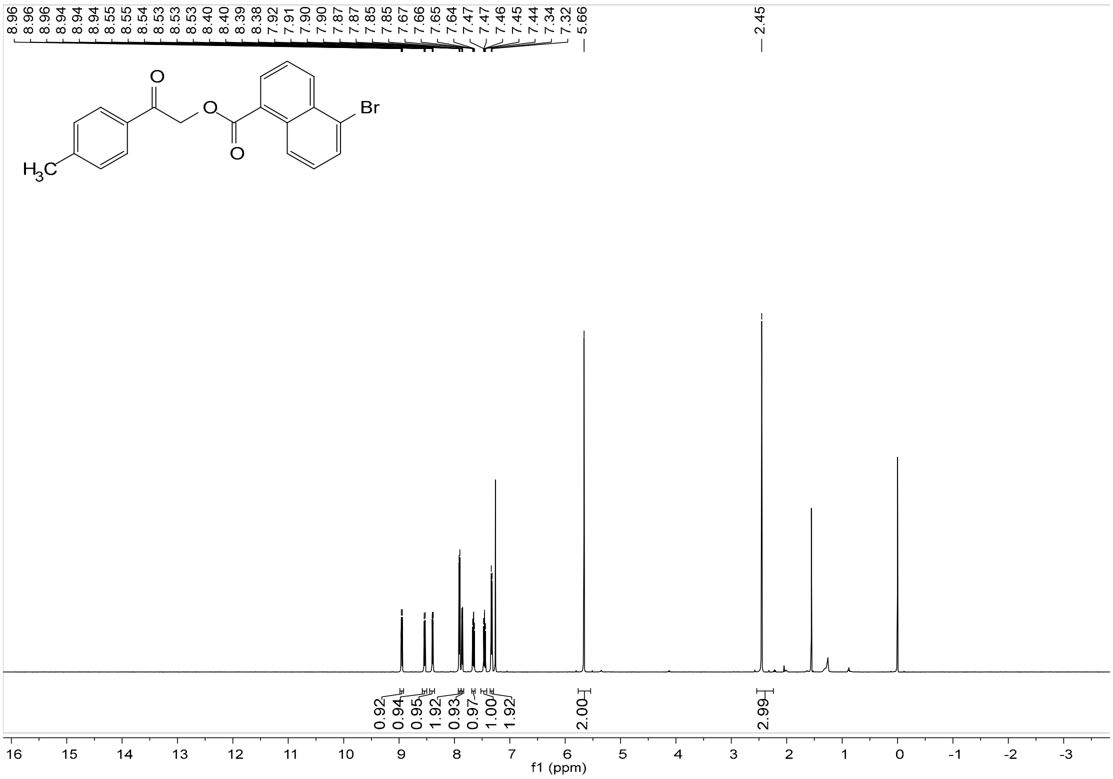

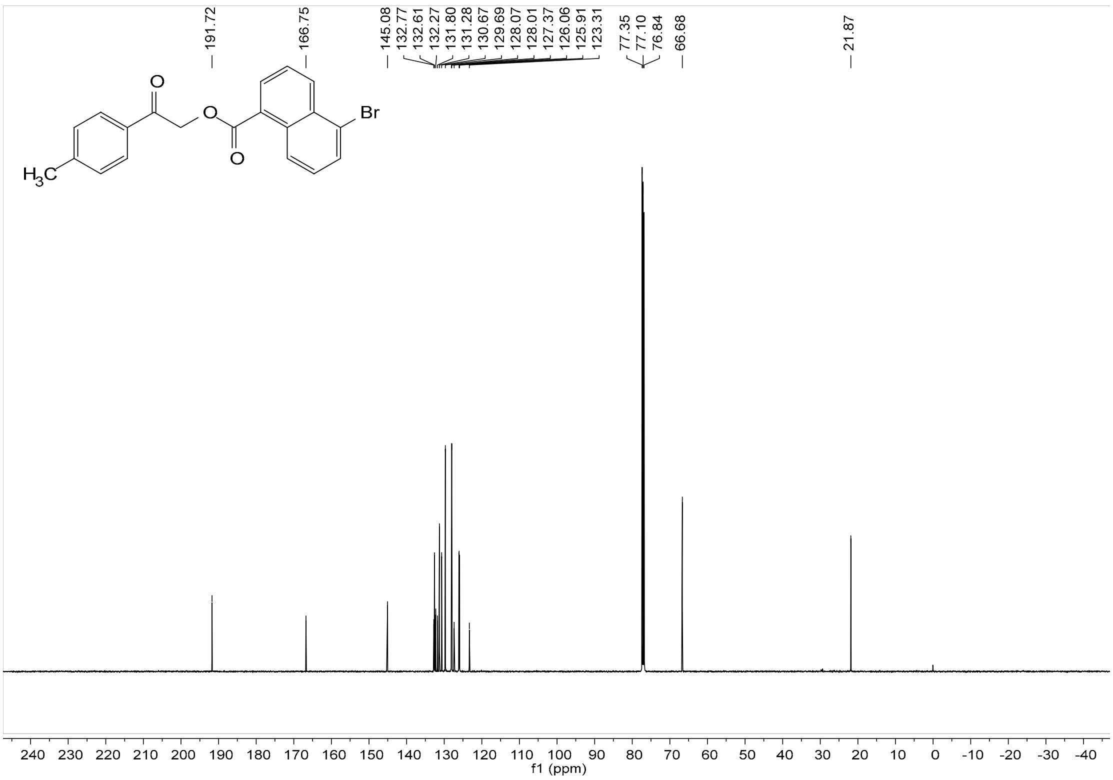

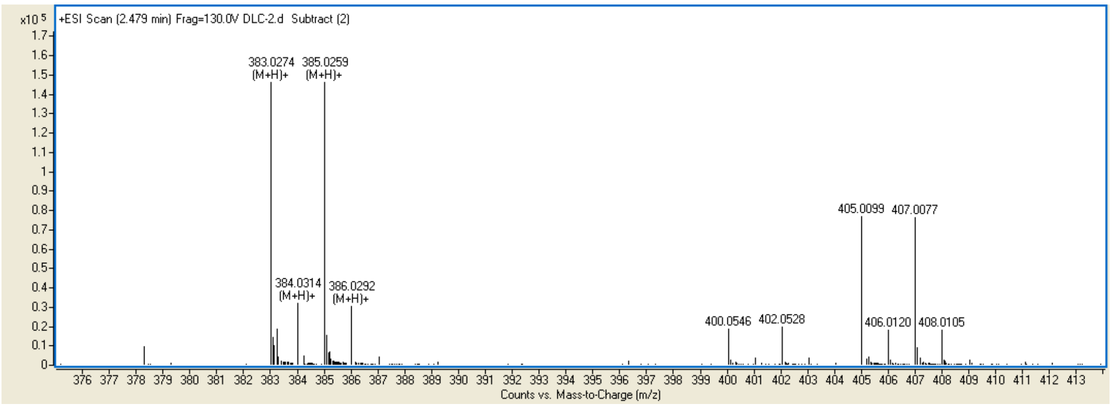

Figure S16. ^{1}H, ^{13}C and HRMS spectra of **C16**

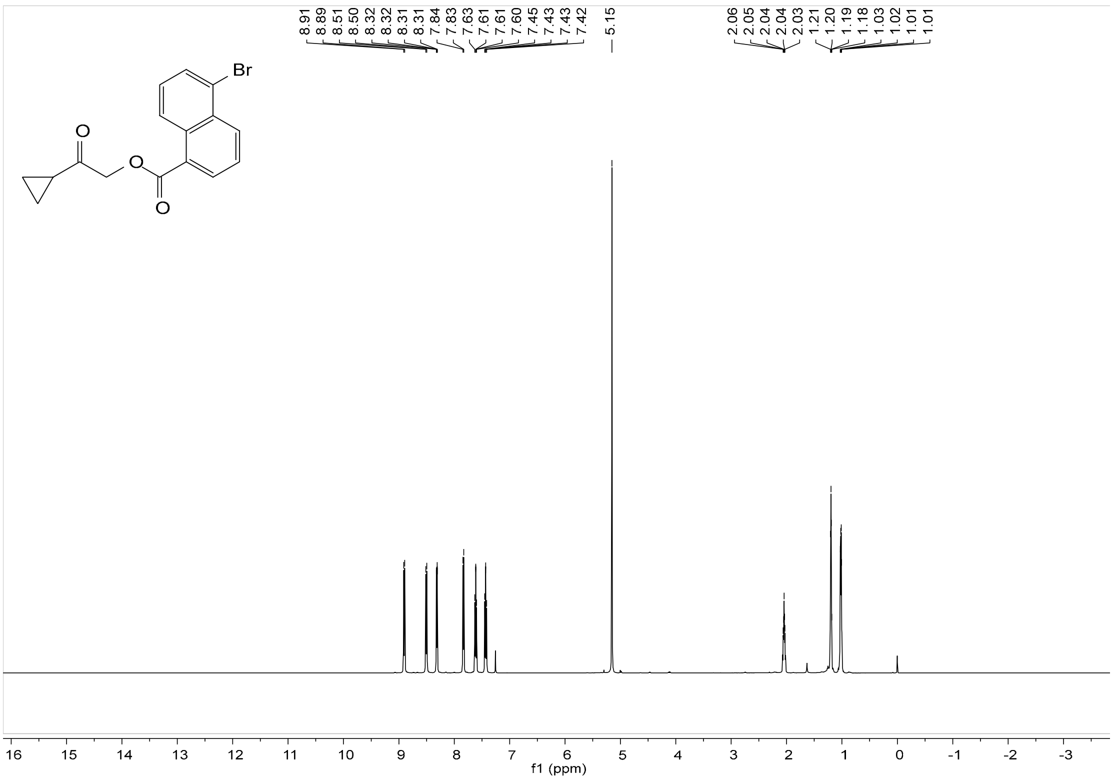
8.91
8.89
8.51
8.50
8.32
8.32
8.31
8.31
7.84
7.83
7.63
7.61
7.61
7.60
7.45
7.43
7.43
7.42
5.15
2.06
2.05
2.04
2.04
2.03
1.21
1.20
1.19
1.18
1.03
1.02
1.01
1.01
Br
O
O
O
f1 (ppm)

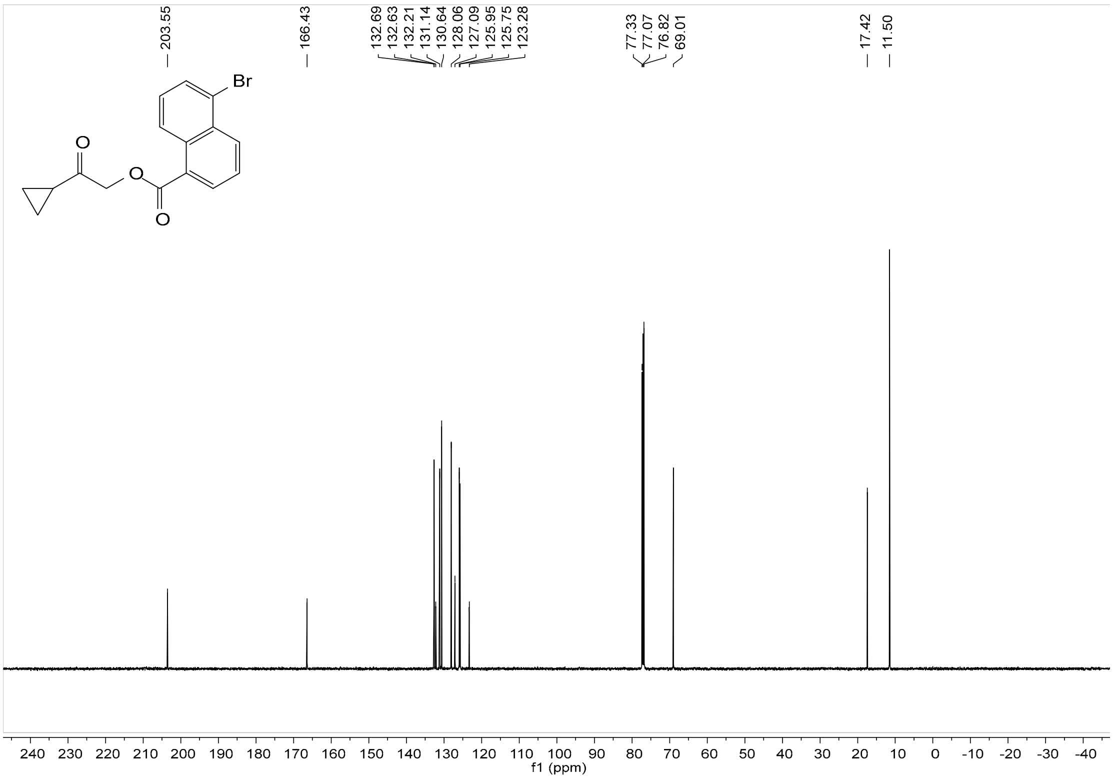
203.55
166.43
132.69
132.63
132.21
131.14
130.64
128.06
127.09
125.95
125.75
123.28
77.33
77.07
76.82
69.01
17.42
11.50
Br
O
O
O
f1 (ppm)

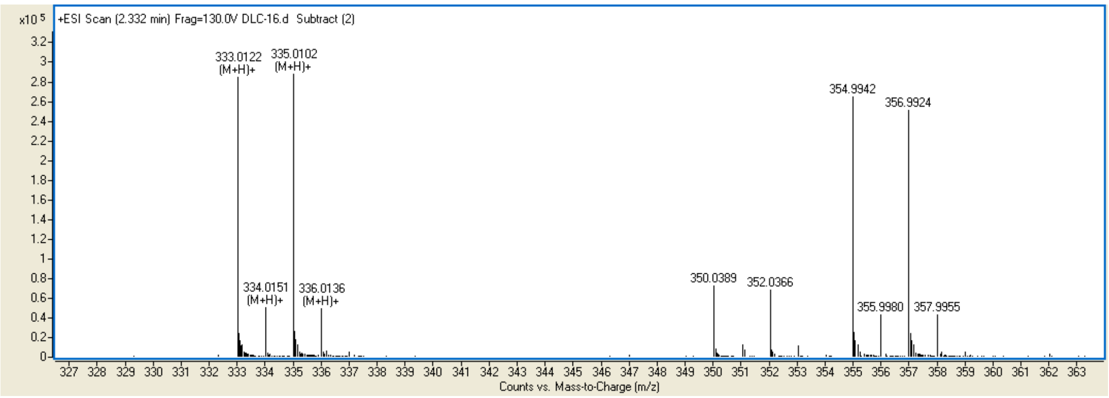

Figure S17. ^{1}H, ^{13}C and HRMS spectra of **C17**

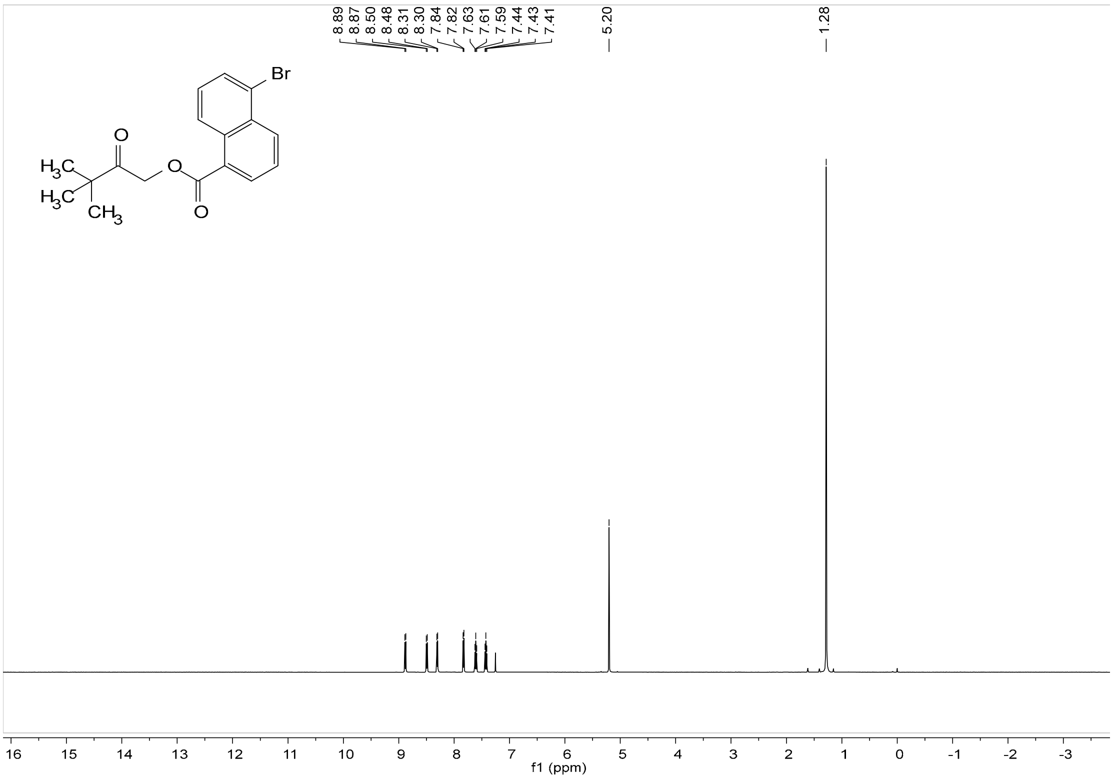

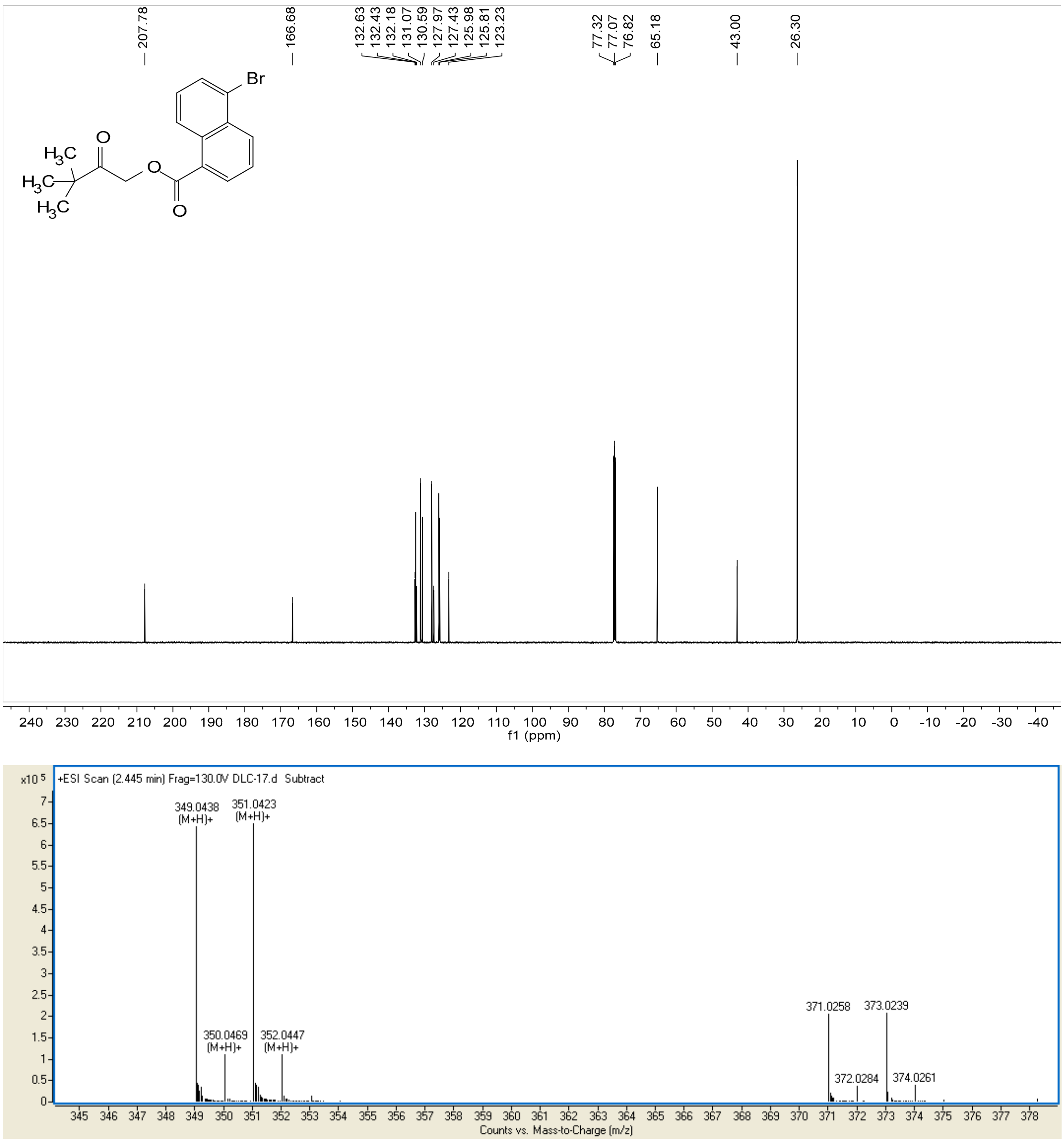

Figure S18. ^{1}H, ^{13}C and HRMS spectra of **C18**

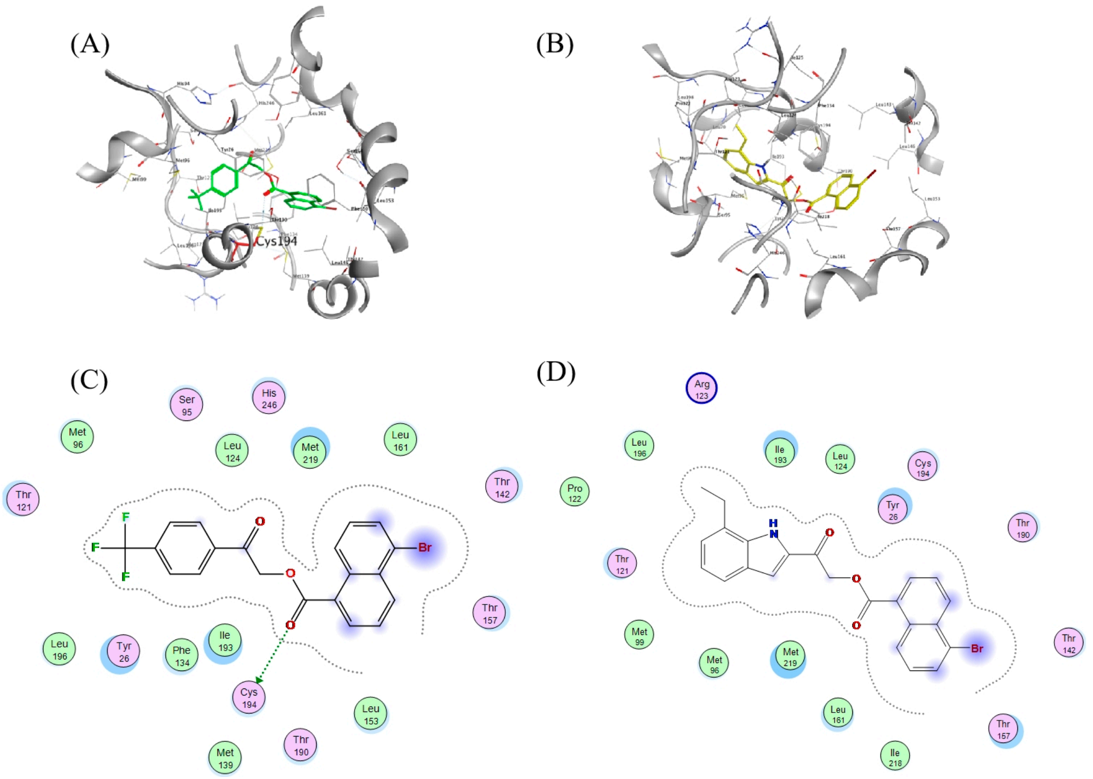

Figure S19. Molecular Docking of C6 and DL1b to ShHTL7 (PBD code:7SNU). The 3D diagram of interaction of the ligands C6 (A) and DL1b (B) with SL receptor ShHTL7. The 2D view of C6 (C) and DL1b (D) in the catalytic site of ShHTL7.

Construction of AtD14 protein expression vector

Wild-type *Arabidopsis* was used for RNA extraction, followed by reverse transcription into cDNA. PCR amplification was performed using primers with the desired vector, with primer sequences as follows: F: CCGGAATTCATGAGTCAACACAACATC, R: CCGCTCGAGAAGTCACCGAGGAAGAGC.

Purification of AtD14 protein expression

(1) The expression strain was inoculated into 5 mL of LB medium containing Kan (1/1000) antibiotic and incubated at 37 °C with shaking at 180 rpm for 8-10 hours.

(2) The well-shaken culture was transferred to 150 mL of LB medium containing Kan (1/1000) antibiotic, and incubated at 180 rpm for 1-2 hours.

(3) 0.2 mM IPTG was added to the culture, inducing protein expression overnight at 18°C with shaking at 180 rpm.

(4) After centrifuging at 5000 rpm for 10 minutes, the supernatant was removed. Then, the pellet was resuspended in 1 ml of binding buffer by thoroughly mixing.

(5) A cell disruptor was used to lyse the cells in ice for 20 minutes and the apply ultrasound at 30% power, 2 seconds on, 3 seconds off. The lysate should be clear and free of cell debris.

(6) Centrifuge at 4°C, 12000 g for 15 minutes and collect the supernatant,

which contains the soluble protein.

(7) 600-700 μl of Ni-affinity column resin was added to the column and washed once with water, washed with binding buffer for twice. The supernatant containing the protein was loaded onto the column, allowing the solution to pass through the gel.

(8) After the solution has passed through, the column was washed twice with 10 mL of binding buffer.

(9) The protein was eluted with 1 mL elution buffer containing 50 mM imidazole and eluted with the elution buffers containing 100 mM and 150 mM imidazole. The eluate was collected in a 1.5 mL centrifuge tube, measuring the protein concentration by collecting 1-2 drops of eluate. After the protein was determined, the desired protein was collected.

(10) The column was washed twice with binding buffer, followed by a wash with water. After the final wash, add 20% ethanol for storage was added for next use (the column can be reused 2-3 times).

(11) SDS-PAGE gel electrophoresis was performed to determine the purity of the purified protein and measure protein concentration.
